# Uncovering the Interplay of Competing Distortions in the Prussian Blue Analogue K_2_Cu[Fe(CN)_6_]

**DOI:** 10.1021/acs.chemmater.2c00288

**Published:** 2022-05-24

**Authors:** John Cattermull, Krishnakanth Sada, Kevin Hurlbutt, Simon J. Cassidy, Mauro Pasta, Andrew L. Goodwin

**Affiliations:** † Department of Chemistry, University of Oxford, Inorganic Chemistry Laboratory, South Parks Road, Oxford OX1 3QR, U.K.; ‡ Department of Materials, University of Oxford, Parks Road, Oxford OX1 3PH, U.K.

## Abstract

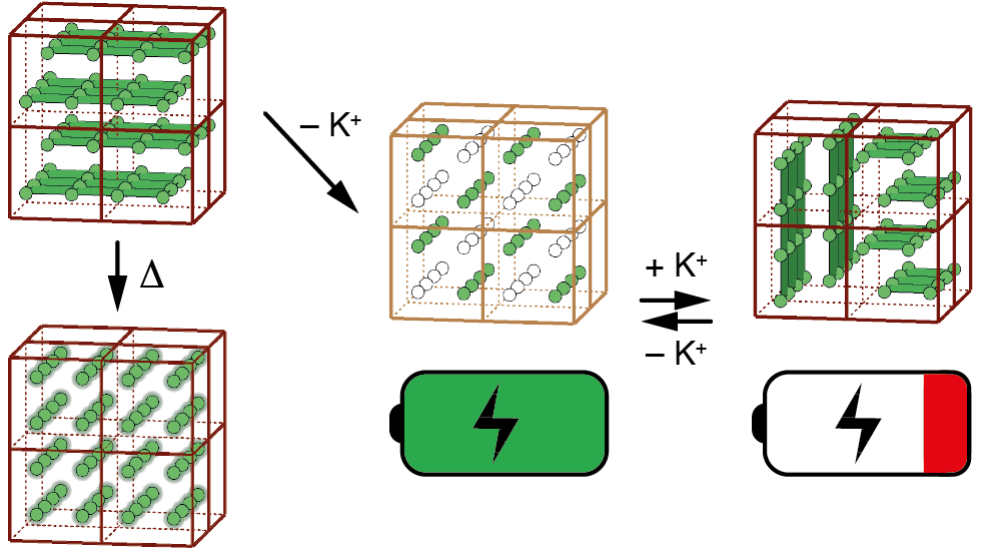

We report the synthesis, crystal structure, thermal response, and electrochemical behavior of the Prussian blue analogue (PBA) K_2_Cu[Fe(CN)_6_]. From a structural perspective, this is the most complex PBA yet characterized: its triclinic crystal structure results from an interplay of cooperative Jahn–Teller order, octahedral tilts, and a collective “slide” distortion involving K-ion displacements. These different distortions give rise to two crystallographically distinct K-ion channels with different mobilities. Variable-temperature X-ray powder diffraction measurements show that K-ion slides are the lowest-energy distortion mechanism at play, as they are the only distortion to be switched off with increasing temperature. Electrochemically, the material operates as a K-ion cathode with a high operating voltage and an improved initial capacity relative to higher-vacancy PBA alternatives. On charging, K^+^ ions are selectively removed from a single K-ion channel type, and the slide distortions are again switched on and off accordingly. We discuss the functional importance of various aspects of structural complexity in this system, placing our discussion in the context of other related PBAs.

## Introduction

Many of the most important and interesting ceramic perovskites are systems in which there is strong interplay among different types of symmetry-lowering distortions.^1−5^ The manganites are arguably the most famous case, for which orbital, magnetic, lattice, and charge degrees of freedom interact;^6^ this interaction is the key to anomalous physical properties such as colossal magnetoresistance, for example.^7,8^ The concept of hybrid improper ferroelectricity is closely related, whereby carefully chosen structural distortions, each of which preserves inversion symmetry, can nonetheless collectively break inversion symmetry and so drive a bulk ferroelectric response.^9^ We^10^ and others^11,12^ have a particular interest in the extension of these same ideas to molecular perovskites—systems in which at least one of the A, B, or X components of the perovskite ABX_3_ structure type is molecular, rather than atomic.^13^

**Figure 1:**
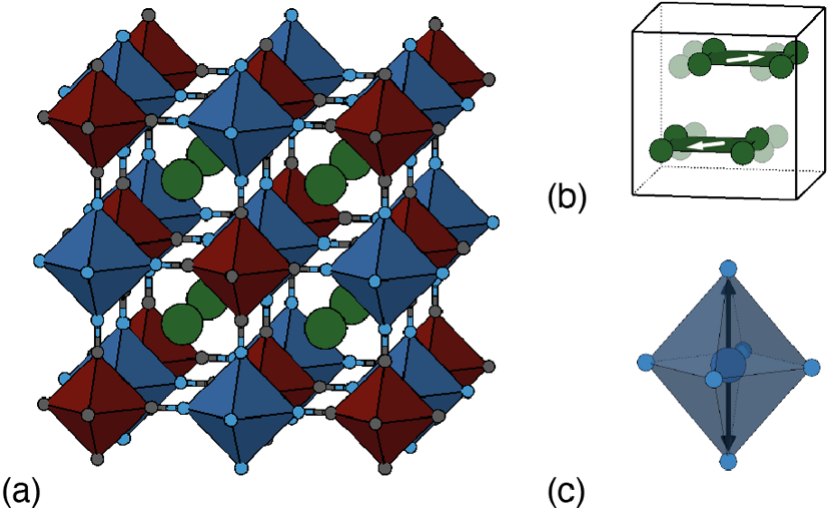
Idealized PBA structure and some common distortions. (a) The structure of low-vacancy PBAs (formula A_*x*_P[R(CN)_6_]) is closely related to that of the double perovskites. P (blue) and R (dark-red) transition metals alternate on a cubic lattice and are connected via P–NC–R links. The A-site cations (green) are situated within the framework cavities. (b) In K-rich PBAs, neighboring layers of K^+^ ions slide in opposite directions along a common ⟨110⟩ axis in order to maximize Coulombic interactions with the anionic framework. (c) Jahn–Teller active P-site cations (e.g., Cu^2+^) can drive cooperative Jahn–Teller order, in which the tetragonal distortion of Cu^2+^ coordination environments aligns along a single common ⟨100⟩ axis.

One key family of molecular perovskites is that of the Prussian blue analogues (PBAs)^14−17^—famous and long-studied systems that are of particular currency in the context of K-ion battery materials (Figure 1a).^18,19^ They are inexpensive to make, employ earth-abundant elements, are accessible through solution-phase synthesis, and benefit from both high operating voltages and favorable charge rates.^20^ It is often considered a key design feature of PBA battery materials that their cubic structure type is relatively unaffected by charge/discharge cycles,^19^ especially in contrast to the substantial anisotropic swelling observed in, e.g., layered cathode materials.^21^ However, it is becoming increasingly clear that PBAs, in fact, harbor a large number of different types of structural distortions.^22,23^ Yet, the implications of these distortions for material function are not fully understood; in particular, do they help or do they hinder?

It was in this context that we sought to prepare and to study the PBA material K_2_Cu[Fe(CN)_6_]: our motivation was the prospect of intentionally introducing a large degree of structural complexity in a material that ought to be electrochemically active. In this way, we might assess the interplay of structural distortions and material function.

We rationalize our choice of composition in the following way. PBAs with high K-ion concentrations on the A-site undergo a “slide” distortion that maximizes Coulombic interactions with the cyanide framework and reduces the cubic PBA symmetry to monoclinic;^22,24^ this distortion occurs in K_2_Mn[Fe(CN)_6_] and K_2_Fe[Fe(CN)_6_], for example, and is analogous to the A-site antipolar distortions of conventional perovskites (Figure 1b).^25,26^ Our second ingredient is the use of Cu^2+^, which introduces a Jahn–Teller instability that ordinarily drives cooperative orbital order and a very different lattice distortion—now tetragonal—as in Cu[Pt(CN)_6_] (Figure 1c).^27,28^ Finally, it is the accessibility of Fe^3+/2+^ electrochemistry that informs our decision to focus on a hexacyanoferrate salt.

Anticipating our results, we will come to show that K_2_Cu[Fe(CN)_6_] does indeed adopt a particularly complex structure (we understand it to be the most complex PBA yet characterized) and at the same time possesses a variety of interesting electrochemical properties. We explore the interplay of these two aspects by using variable-temperature X-ray diffraction measurements, on the one hand, to understand the hierarchy of distortion energy scales at play, and then *ex situ* diffraction measurements during charge/discharge cycles, on the other hand, to relate these distortions to the structural mechanism of K-ion (de)insertion.

## Methods

### Synthesis

On the basis of the exploration of synthesis parameters reported in ref (25), we synthesized polycrystalline samples of K_2_Cu[Fe(CN)_6_] via a citrate-assisted precipitation in aqueous media. CuNO_3_·3H_2_O (Sigma-Aldrich, 1 mmol) was dissolved in an aqueous solution of potassium citrate (Sigma-Aldrich, 1 M, 20 mL). This solution was added dropwise to a stoichiometric aqueous solution of K_4_Fe(CN)_6_ (Sigma-Aldrich, 20 mL) at 80 °C with stirring. The mixture was stirred for 2 h and then allowed to age for a further 2 h. During this period, a deep-red precipitate formed. This precipitate was isolated by centrifugation and washed with a 50:50 water/ethanol mixture in order to prevent the solid dispersing. The solid was dried in air at 70 °C.

### Materials Characterization

Elemental composition was determined by inductively coupled plasma mass spectrometry (ICP-MS) (Shimadzu ICPMS-2030), and water content was estimated using thermogravimetric analysis (TGA) (NETZSCH STA 449 F3 Jupiter) under Ar at a heating rate of 5 °C min^–1^. Scanning electron microscopy (SEM) was carried out on a Zeiss Merlin microscope. Synchrotron X-ray diffraction (XRD) measurements were performed on I11 beamline of the Diamond Light Source operating with an X-ray wavelength of 0.826872 Å. The position-sensitive detector was used to collect diffraction patterns over the temperature range 30–450 °C with a hot-air blower. *Ex situ* X-ray powder diffraction measurements of the electrode materials were performed using a Rigaku Smartlab diffractometer (Cu Kα). All Rietveld and Pawley refinements were carried out using the TOPAS-Academic software.^29^

### Electrochemical Characterization

Electrodes were prepared by mixing 70 wt % active material, 20 wt % carbon black (Super P), and 10 wt % poly(vinylidene fluoride) (PVDF) in a mortar and pestle with 1-methyl-2-pyrrolidone (NMP) to form a slurry. The slurry was pasted on carbon cloth (Fuel Cell Store ELAT hydrophilic carbon cloth) with a mass loading of around 10 mg cm^–2^, dried in air, and then dried overnight at 80 °C under vacuum. Electrochemical measurements were performed in flooded three-electrode cells sealed under Ar atmosphere in an aqueous solution of K_2_SO_4_ (0.5 M) acidified to pH 1.8 with H_2_SO_4_. A Hg/Hg_2_SO_4_ reference in saturated K_2_SO_4_ and a Pt counter electrode were used.

### Computational Methods

Density-functional theory (DFT) calculations were performed using the Vienna Ab initio Simulation Package (VASP).^30,31^ Candidate structures were relaxed using the HSE06 functional.^32,33^ All calculations were Γ-point only with a planewave kinetic-energy cutoff of 520 eV. Electronic and ionic convergence criteria were 10^–5^ eV and 0.05 eV Å^–1^, respectively.

## Results and Discussion

### Preparation and Characterization of K_2_Cu[Fe(CN)_6_]

The chemical composition of our K_2_Cu[Fe(CN)_6_] sample, prepared as described above, was determined using ICP-MS measurements. ICP provides a robust measure of both Fe and Cu content, but is notoriously unreliable in determining potassium content,^34^ which must be deduced by consideration of charge balance. We found the Fe/Cu ratio to be 0.979(8). The degree of hydration was estimated to be 0.11 on the basis of the mass loss observed in TGA measurements, although some of this will be surface-absorbed water. Collectively these measurements implied a composition of K_1.96_Cu[Fe(CN)_6_]_0.98_·0.11H_2_O; we use the simplified approximate formula K_2_Cu[Fe(CN)_6_] hereafter.

**Figure 2:**
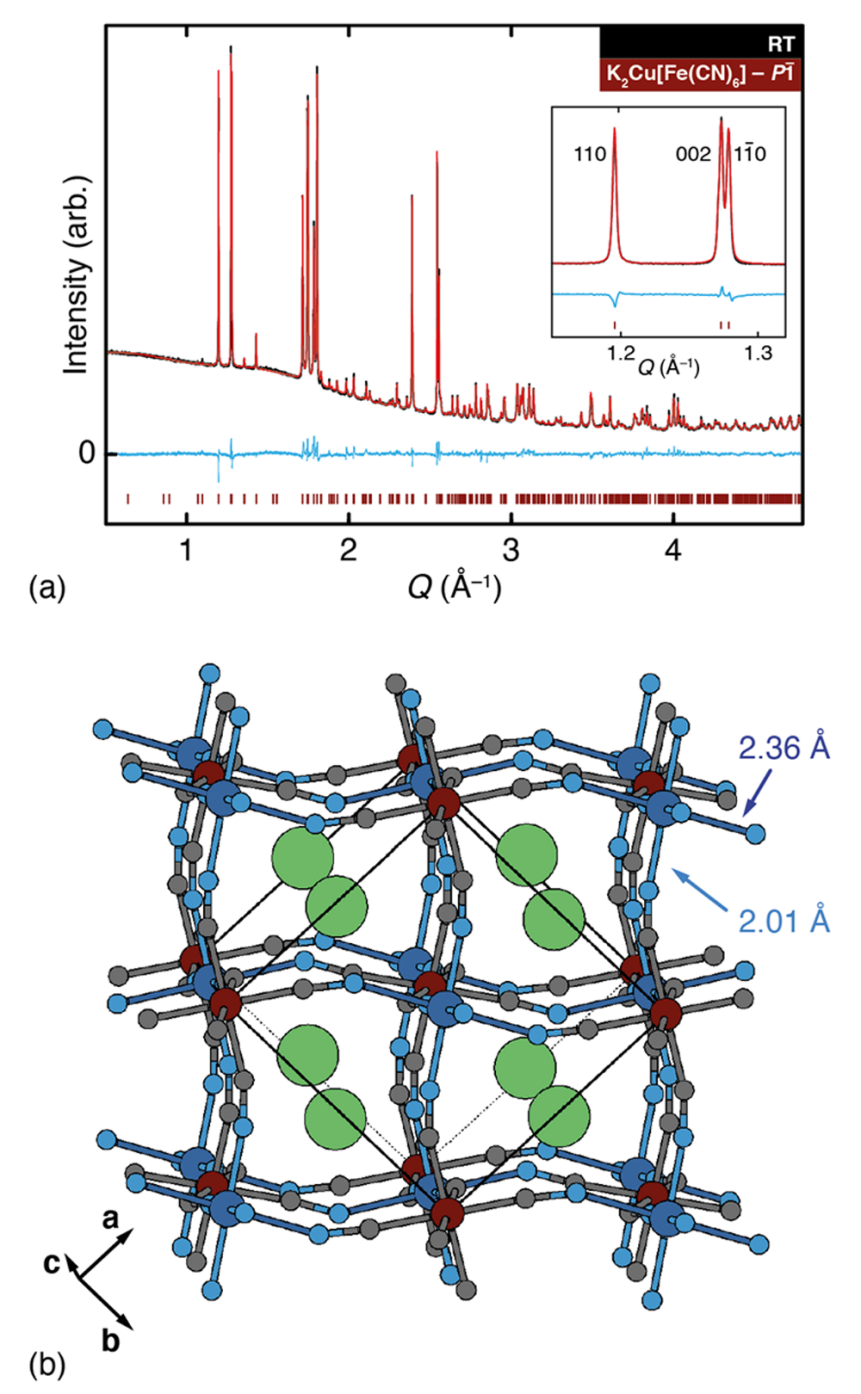
(a) Rietveld fit to the room-temperature synchrotron X-ray powder diffraction pattern of K_2_Cu[Fe(CN)_6_] with data (black), fit (red), difference function (blue), and calculated reflection positions (dark-red tick marks). The inset shows a representative low-angle region of the pattern in which the triclinic splitting is very obvious—here between the 110 and 11̅0 reflections. (b) Representation of the final structural model determined from our refinements. K atoms are shown in green, Cu in dark blue, Fe in dark red, C in gray, and N in light blue. Note the presence of large-scale K-ion off-centering. The Cu–N bond lengths partition into “short” and “long” bonds, shown here as light and dark blue cylinders, respectively. The arrangement of the different Cu–N bonds reflects collective Jahn–Teller order, with Cu^2+^ octahedra elongated along a direction close to [110].

The ambient-temperature synchrotron X-ray diffraction pattern of K_2_Cu[Fe(CN)_6_] is shown in Figure 2a. The diffraction profile is surprisingly different to that of the monoclinic PBAs, such as K_2_Mn[Fe(CN)_6_];^25^ in particular, the very strongest low-angle reflections show further peak splitting than allowed even in the already-low-symmetry monoclinic structure type (see inset to Figure 2a). Using the distortion mode refinement approach implemented within TOPAS,^29^ we obtained a structure solution in the triclinic space-group *P*1̅ with an excellent fit-to-data (*R*_wp_ = 1.95%). We came to rationalize this particularly low-symmetry structure in terms of competing distortion modes. Details of the structural model are given in Table 1, and the structure itself is illustrated in Figure 2b; our refinement protocol is discussed in more detail in the Supporting Information (SI).

**Table 1:** Crystallographic Parameters for the *P*1̅ Structure of K_2_Cu[Fe(CN)_6_] at Ambient Temperature^a^

*a*/Å	7.0560(5)
*b*/Å	7.3401(6)
*c*/Å	9.8698(6)
α/°	89.8890(10)
β/°	89.9083(11)
γ/°	86.154(10)
*V*/Å^3^	510.025(2)
*Z*	2

Atom	*x*	*y*	*z*	*B*_eq_/Å^2^
K1	–0.0378(5)	0.5655(5)	0.2495(7)	2.9(2)
K2	0.4917(5)	0.0548(5)	0.2519(7)	4.5(2)
Cu1	0	0	0.5	1.01(3)
Cu2	0.5	0.5	0	1.01
Fe1	0	0	0	1.01
Fe2	0.5	0.5	0.5	1.01
C1	0.763(3)	0.874(3)	0.0248(16)	1.01
C2	0.139(3)	0.773(3)	*-*0.0241(18)	1.01
C3	0.360(3)	0.294(3)	0.5387(16)	1.01
C4	0.735(3)	0.364(3)	0.4777(18)	1.01
C5	0.074(3)	0.005(3)	0.181(2)	1.01
C6	0.571(3)	0.537(3)	0.683(2)	1.01
N1	0.636(2)	0.781(2)	0.0381(14)	1.01
N2	0.255(2)	0.659(2)	*-*0.0095(16)	1.01
N3	0.265(2)	0.168(2)	0.5509(14)	1.01
N4	0.846(2)	0.245(2)	0.4878(16)	1.01
N5	0.036(2)	–0.006(2)	0.297(2)	1.01
N6	0.543(2)	0.524(2)	0.799(2)	1.01

a In our Rietveld refinements we allowed K occupancies to vary from unity, obtaining the values 0.946(13) and 0.986(13) for K1 and K2. The *B*_eq_ values for all non-K atoms were constrained to be the same in order to reduce the number of independent variables.

Despite the low symmetry of this structure, our use of high-resolution synchrotron X-ray diffraction measurements has allowed us to obtain sensible atomic coordinates. For example, we find that the octahedral coordination geometry of the hexacyanoferrate groups is well preserved, and that even the C and N positions are reasonable despite the poor scattering contrast of these light elements in the presence of K, Fe, and Cu. Importantly, the structural distortions we intended to introduce by choosing the K_2_Cu[Fe(CN)_6_] composition are evident in this structure solution. For example, the K atoms have displaced from their high-symmetry positions by about 0.5 Å to give precisely the same type of slide distortion seen in other K-rich PBAs (albeit that the magnitude of distortion is particularly large here). Likewise, of the six distinct Cu–N bond lengths, two are significantly longer than the other four (2.36 Å vs 2.02 Å), as expected for a Jahn–Teller-distorted octahedral Cu^2+^ coordination environment.^1,28^ Cooperative tilting of the transition-metal coordination polyhedra is also observed; the particular tilt system is given by the Glazer notation^35^*a*^0^*a*^0^*c*^+^ and is the simplest tilt distortion compatible with the K-ion slides we have observed.^22,36^ An important feature of this structure is the existence of two symmetry-inequivalent K^+^ sites, a point that is discussed later in this paper. Further details of key bond lengths and coordination environments are given in the SI.

### Distortion-Mode Analysis

In general, one ought to be skeptical of low-symmetry structure solutions, so it is natural to question if there a logical reason why the crystal structure of K_2_Cu[Fe(CN)_6_] is triclinic.

We argue first by comparing against the known structure of K_2_Mn[Fe(CN)_6_], which has the monoclinic *P*2_1_/*n* space-group symmetry common to many K-rich PBAs.^25^ Formally, this monoclinic structure type is related to the idealized cubic PBA parent structure (*Fm*3̅*m* symmetry) by the combined activation of the slide distortion shown in Figure 1b and a cooperative *a*^0^*a*^0^*c*^+^ octahedral tilt of the framework structure that always seems to accompany it.^22,23^ The former deformation transforms as the X_5_^+^ irreducible representation (irrep; note that we are using labels relative to the double-perovskite *Fm*3̅*m* parent with B-site ions located at the cell origin) and the latter as X_3_^+^; it is the interplay of these two distortion modes that reduces the PBA symmetry to *P*2_1_/*n*.^22^ Replacing Mn^2+^ by the Jahn–Teller-active Cu^2+^ understandably leads to an additional distortion of the type illustrated in Figure 1c, which transforms as Γ_3_^+^. We find by using the ISOTROPY software^37,38^ that this additional distortion reduces the crystal symmetry from *P*2_1_/*n* to *P*1̅, with the same cell orientation as observed in our Rietveld refinement. Consequently, adding collective Jahn–Teller order to the monoclinic K_2_Mn[Fe(CN)_6_] structure type necessarily implies triclinic symmetry.

A related argument can be made by considering the structure of RbCu[Co(CN)_6_].^39^ This system has orthorhombic *Cccm* space-group symmetry, which is understood as arising from the interplay of the collective Jahn–Teller order of Cu^2+^ ions (again, Γ_3_^+^) with either the *a*^0^*a*^0^*c*^+^ tilt distortion (X_3_^+^) or “rodlike” Rb cation order (X_4_^+^). The group theory result is that any two of these three distortion types necessarily gives the third, so there is no way of telling from symmetry arguments alone which two of these three are physically responsible for symmetry lowering. Whatever the case, there is no off-centering of the Rb^+^ ions in this orthorhombic structure. Replacing Rb^+^ by the smaller K^+^ and doubling the A-site cation content introduces the X_5_^+^ slide distortion; again, ISOTROPY analysis indicates that this additional distortion lowers the crystal symmetry to *P*1̅, as observed experimentally.

**Figure 3:**
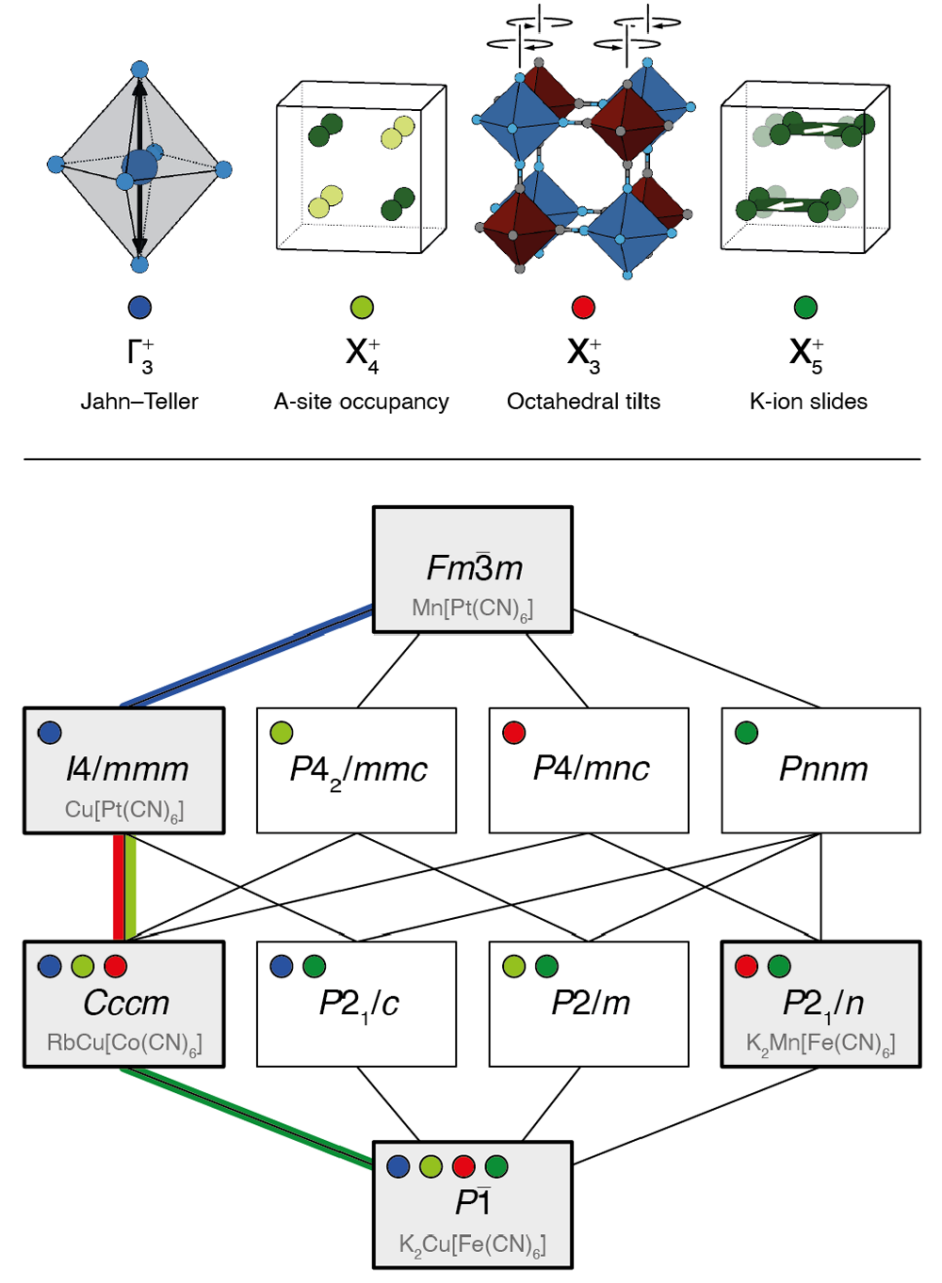
Symmetry relationships in distorted PBAs. The four key distortion types relevant to our study are (top, left to right) cooperative Jahn–Teller order, “rodlike” A-site cation order, *a*^0^*a*^0^*c*^+^ octahedral tilts, and K-ion slides. Starting from the aristotypic double-perovskite, the symmetry map at the bottom of the figure represents the space-group symmetry that results from successive activation of each distortion type. Note that any two of the Γ_3_^+^, X_4_^+^, and X_3_^+^ distortions necessarily activates the third. Combinations with known PBA exemplars are highlighted in gray. The path between *P*1̅ and *Cccm* structure types, which is key to the thermal and electrochemical response of K_2_Cu[Fe(CN)_6_], is highlighted in green as it corresponds to activation or deactivation of the K-ion slide distortion. We have used the space-group labels *P*2_1_/*c* and *P*2_1_/*n* for two of the distorted structure types to convey the point that the resulting structures are inequivalent: while either might be transformed to the other space-group setting, the unique axis is different in the two cases.

We illustrate these various symmetry relationships in Figure 3, where we draw on the established visual language used to relate progressively complex tilt distortions in conventional perovskites.^36,40,41^ The key point is that one can consider the low-symmetry *P*1̅ structure we observe as the inevitable consequence of introducing either cooperative Jahn–Teller order into the monoclinic K-rich PBA structure type or K-ion-driven slides into the orthorhombic Jahn–Teller-ordered structure.

Just as the *Cccm* structure of RbCu[Co(CN)_6_] contains two crystallographically distinct A-site environments—in that case, one empty and the other occupied by Rb^39^—so is it the case that there are two distinct K environments in our new *P*1̅ structure of K_2_Cu[Fe(CN)_6_] (as noted above). The authors of ref (42) argued on the basis of Madelung constants that the resulting “rodlike” Rb order has a physical basis, but our instinct is that there is no strong chemical driving force for this distinction in a system such as K_2_Cu[Fe(CN)_6_] where all A sites are occupied. Instead the existence of two K-ion sites is a consequence of symmetry-breaking by other structural distortions with more obvious physical origins.

### Density Functional Theory Calculations

We used density functional theory (DFT) calculations as a further check on the validity of our structural model for K_2_Cu[Fe(CN)_6_]. Starting from the lattice parameters and atomic coordinates determined in our Rietveld refinement, the crystal structure was fully relaxed using the HSE06 functional to account for strong electronic correlation.^43^ The relaxed unit cell dimensions are listed in Table 2 and differ by less than 1% from our experimental values. Individual atomic coordinates also showed relatively small deviations. The Fe and Cu atom positions do not vary, the K atoms shifted with a root-mean-squared (r.m.s.) displacement of 0.09 Å, and the C and N atoms showed the largest shifts with r.m.s. displacements of 0.18 and 0.20 Å, respectively. Given the difficulty of refining C and N positions in the presence of electron-rich elements from powder X-ray diffraction data, we consider this difference entirely reasonable. Importantly, all of the distortion modes identified above—K-ion slides, collective Jahn–Teller order, and octahedral tilts—were evident also in this relaxed DFT structure. For completeness, the DFT atomic coordinates are given in the SI.

**Table 2:** DFT (0 K) Unit Cell Parameters for K_2_Cu[Fe(CN)_6_] and the Corresponding Differences Relative to Our Experimental Values Measured at 295 K

parameter	DFT	experiment	difference (%)
*a*/Å	7.092	7.0560(5)	0.51
*b*/Å	7.335	7.3401(6)	0.07
*c*/Å	9.842	9.8698(6)	0.28
α/°	89.713	89.8890(10)	0.20
β/°	89.990	89.9083(11)	0.01
γ/°	85.520	86.154(10)	0.74

### High-Temperature Behavior

**Figure 4:**
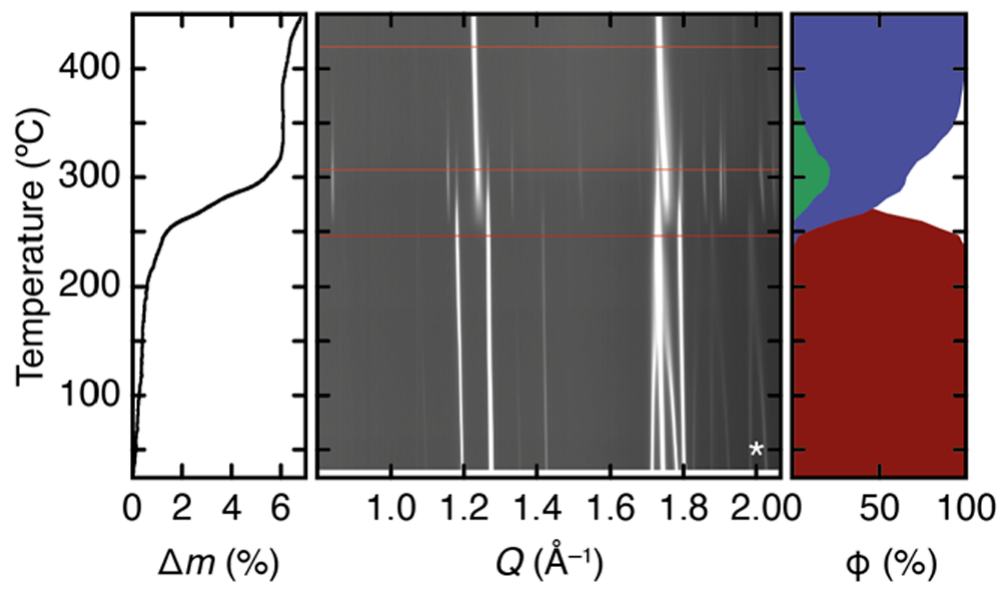
Variable-temperature structural response of K_2_Cu[Fe(CN)_6_]. The temperature dependence of the relative mass loss *Δm* measured using TGA, a representative section of the X-ray powder diffraction pattern, and the phase fractions ϕ obtained using constrained Rietveld refinements to the X-ray data are displayed left to right. Here, dark red corresponds to the ambient K_2_Cu[Fe(CN)_6_] phase, green to the transient decomposition product KCu(CN)_2_, and dark blue to K_2_Fe[Fe(CN)_6_]. The three horizontal lines drawn on the diffractogram correspond to the data sets shown in Figure 5.

In order to determine the hierarchy of distortion energy scales in K_2_Cu[Fe(CN)_6_], we sought to characterize its behavior on heating. After all, the thermal response of a material is dominated by the activation of the lowest-energy deformations.^44^ We first used TGA to understand the compositional stability of the system; our results are shown in Figure 4. Three regimes are evident. First, heating to ∼250 °C sees the loss of a small amount of surface and structural water, as is common for PBAs in general.^45^ A more substantive mass loss event occurs around 250–335 °C, resulting in a solid that eventually decomposes above 425 °C.

Our variable-temperature synchrotron X-ray powder diffraction measurements focus on the temperature range 30–450 °C and are consistent with the TGA findings (Figure 4). The ambient *P*1̅ phase persists from room temperature until ∼250 °C. Within this regime a number of peaks coalesce and others disappear, suggesting a continuous ascent in symmetry (see, for example, the pair of peaks marked with an asterisk in Figure 4). On heating above 250 °C, the ambient phase is progressively lost, and two new phases grow in. One appears then disappears, and the other remains the dominant phase through the highest temperatures explored in our measurements. The diffraction pattern of this persistent phase appears close to that of a conventional cubic PBA for the highest temperatures probed in our measurements.

**Figure 5:**
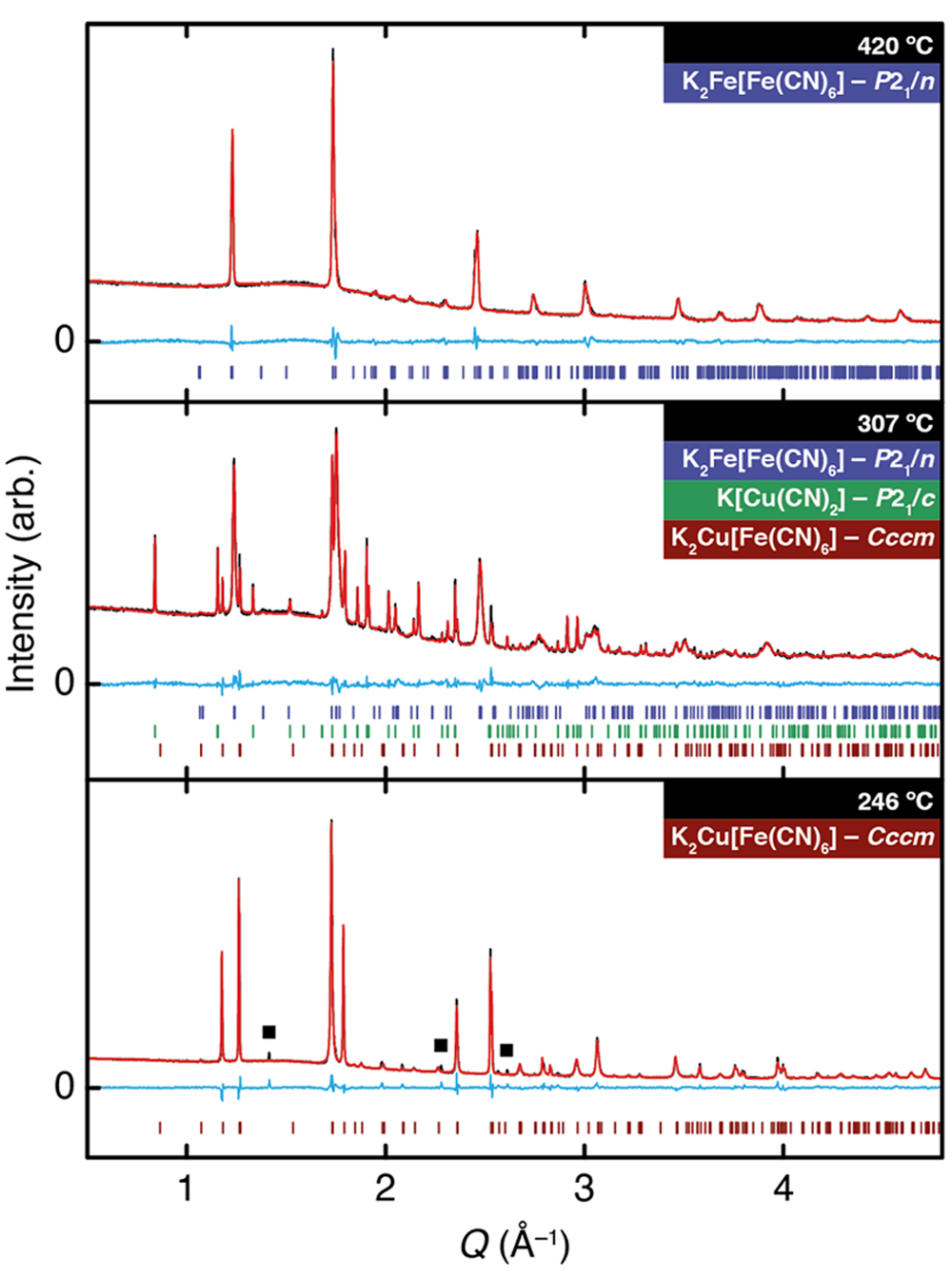
Constrained Rietveld fits to the X-ray powder diffraction patterns measured for our K_2_Cu[Fe(CN)_6_] sample heated to 420 °C (top), 307 °C (middle), and 246 °C (bottom). The first of these corresponds to K_2_Fe[Fe(CN)_6_] at the point that its monoclinic distortion effectively vanishes, the second corresponds to the temperature at which all three crystalline phases are present, and the third corresponds to the first point at which the structure of K_2_Cu[Fe(CN)_6_] is better described by *Cccm* than *P*1̅ space-group symmetry. The few weak peaks remaining that are forbidden in *Cccm* are indicated by filled squares.

Focusing first on the thermal behavior of the ambient *P*1̅ phase, we carried out a series of sequential distortion-mode Rietveld refinements for the diffraction patterns measured at each temperature over the range 30–350 °C. We found the very strongest variation in distortion-mode amplitudes for those distortions related to the K-ion slide distortion (see SI). In fact, by 250 °C, the lattice strain associated with this distortion (Γ_5_^+^ irrep) has essentially vanished such that the diffraction pattern of K_2_Cu[Fe(CN)_6_] at this temperature is actually better described in the orthorhombic space-group *Cccm* than in *P*1̅. We show a fit to the data using this higher-symmetry space group in Figure 5. The microscopic picture that emerges is that K-ion displacements are most easily activated on heating, such that temperature switches off the slide distortion and its symmetry-lowering effect—all that remains are the distortions found in related systems with larger A-site cations (e.g., RbCu[Co(CN)_6_]).^39^

While the focus of our study is on the ambient *P*1̅ phase, we were nevertheless interested to understand in general terms the decomposition process. In this spirit, we were able to match the diffraction profile of the high-temperature transient phase to that of potassium dicyanocuprate(I), KCu(CN)_2_.^46^ The cyanide ion is well known to reduce copper(II) to copper(I),^47,48^ so the emergence of this phase implies the breaking of Fe–CN bonds at this elevated temperature. KCu(CN)_2_ is understood to melt at around 290 °C, which is presumably why the diffraction pattern of this phase disappears on further heating. One possible decomposition pathway for K_2_Cu[Fe(CN)_6_] is the reaction
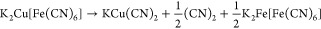
The mass loss observed in our TGA measurements is broadly consistent with that expected for cyanogen evolution (see SI). Moreover, we find the second, persistent, high-temperature phase to be well modeled by the *P*2_1_/*n* structure of K_2_Fe[Fe(CN)_6_];^26^ the monoclinic distortion in this phase decreases with increasing temperature such that it has essentially vanished by 425 °C, and the structure is almost cubic. Key corresponding Rietveld fits are shown in Figure 5, and the associated phase fractions are given in panel (a) of the same figure.

Our variable-temperature X-ray diffraction results show that K_2_Cu[Fe(CN)_6_] responds to heating by first unwinding the K-ion slide distortion and then, we propose, by exsolving Cu^2+^, which is reduced by free cyanide to give KCu(CN)_2_ as a transient solid phase and the thermally robust PBA K_2_Fe[Fe(CN)_6_]. Of course, it is possible that some Cu remains in this final PBA—our X-ray measurements would be insensitive to Cu/Fe compositions—but the space-group symmetry rules out any cooperative Jahn–Teller distortion. Other decomposition mechanisms may be equally consistent with our data, and a definitive investigation is beyond the scope of this study. As a final point, we note that not only are the K-ion slides the key thermally activated distortion in this material, but also that the observed transition to *Cccm* implies it is probably right to think of them as a fundamental distortion in their own right and not simply a byproduct of other distortions, such as tilts.^23^

### Electrochemistry

**Figure 6:**
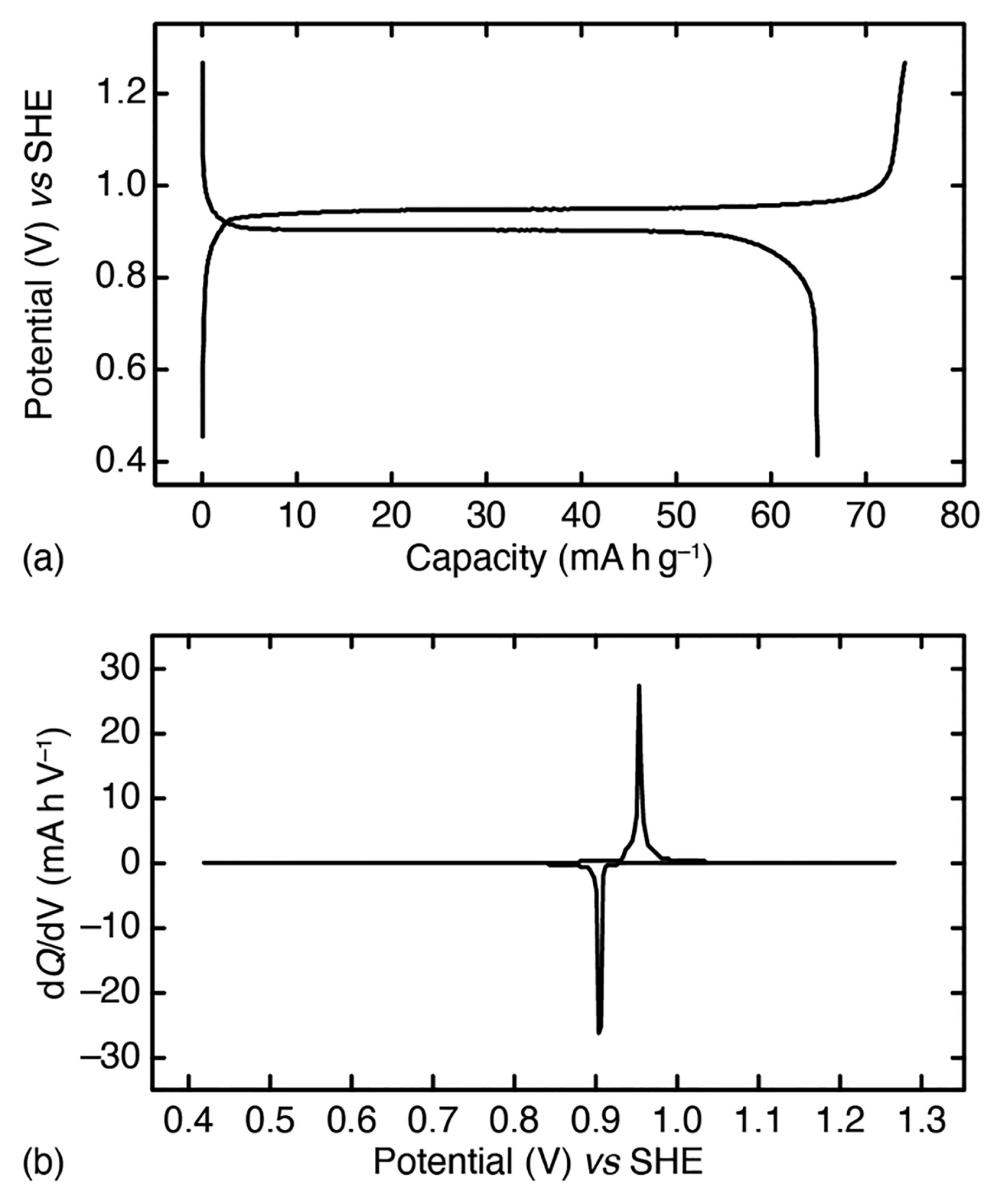
Electrochemical characterization of K_2_Cu[Fe(CN)_6_]. (a) The galvanostatic cycle measured at a cycling rate of C/6 shows a maximum specific capacity of 73.8 mA h g^–1^ centered on 0.949 V. The flat profile is characteristic of a two-phase mechanism. (b) The corresponding differential capacity function.

We turn now to the electrochemistry of K_2_Cu[Fe(CN)_6_], with a particular emphasis on understanding its structural response to K-ion (de)insertion. For our electrochemical measurements, we used an aqueous cell setup designed to perform well at high operating potentials; the linear sweep voltammetry (LSV) shows good stability of the electrolyte in the upper potential limit of 1.265 V versus the standard hydrogen electrode (SHE) (see SI). Our results, obtained using a cycling rate of C/6, are shown in Figure 6a. The material cycles at a high voltage of 0.949 V versus SHE at the midcomposition on charging, with a capacity of 73.8 mA h g^–1^ on the first charge. This capacity is very close to the theoretical value for a vacancy-free and anhydrous K_2_Cu[Fe(CN)_6_] composition (75.8 mA h g^–1^), which is further evidence of the low-vacancy/high-potassium content of our sample. Not all capacity is recovered on subsequent discharge.

The profile of the galvanostatic cycle is characteristic of a biphasic reaction: there is a plateau in the potential measured that corresponds to a sharp peak in the differential capacity function (Figure 6b). A two-phase mechanism is also supported by the potentiostatic intermittent titration technique (PITT), which shows the characteristic bell-shaped *I*–*t* curve that arises from a delayed response of the current following each step in voltage (see SI).

There is an interesting comparison to be drawn between the behavior we observe for K_2_Cu[Fe(CN)_6_] and that of the closely related and well-established cathode material K_0.71_Cu[Fe(CN)_6_]_0.72_.^18^ With its large fraction of hexacyanoferrate vacancies, there is no long-range cooperative Jahn–Teller order in the latter; instead, its crystal structure (which is very disordered) has cubic average symmetry. Cubic symmetry is maintained on K-ion insertion/deinsertion—the presence of vacancies in that phase frustrating long-range order of any local distortions.^22,49^ This is why K_0.71_Cu[Fe(CN)_6_]_0.72_ cycles via a single-phase (solid-solution) mechanism. By contrast, we expect that the K-ion slide distortion in the vacancy-free K_2_Cu[Fe(CN)_6_] will be switched off at a critical potassium content because K^+^ ions are removed during charge.^22,25^ On the basis of the symmetry relationships shown in Figure 3, one anticipates a transition to the *Cccm* structure type at such a point, which would explain the two-phase mechanism we observe here.

**Figure 7:**
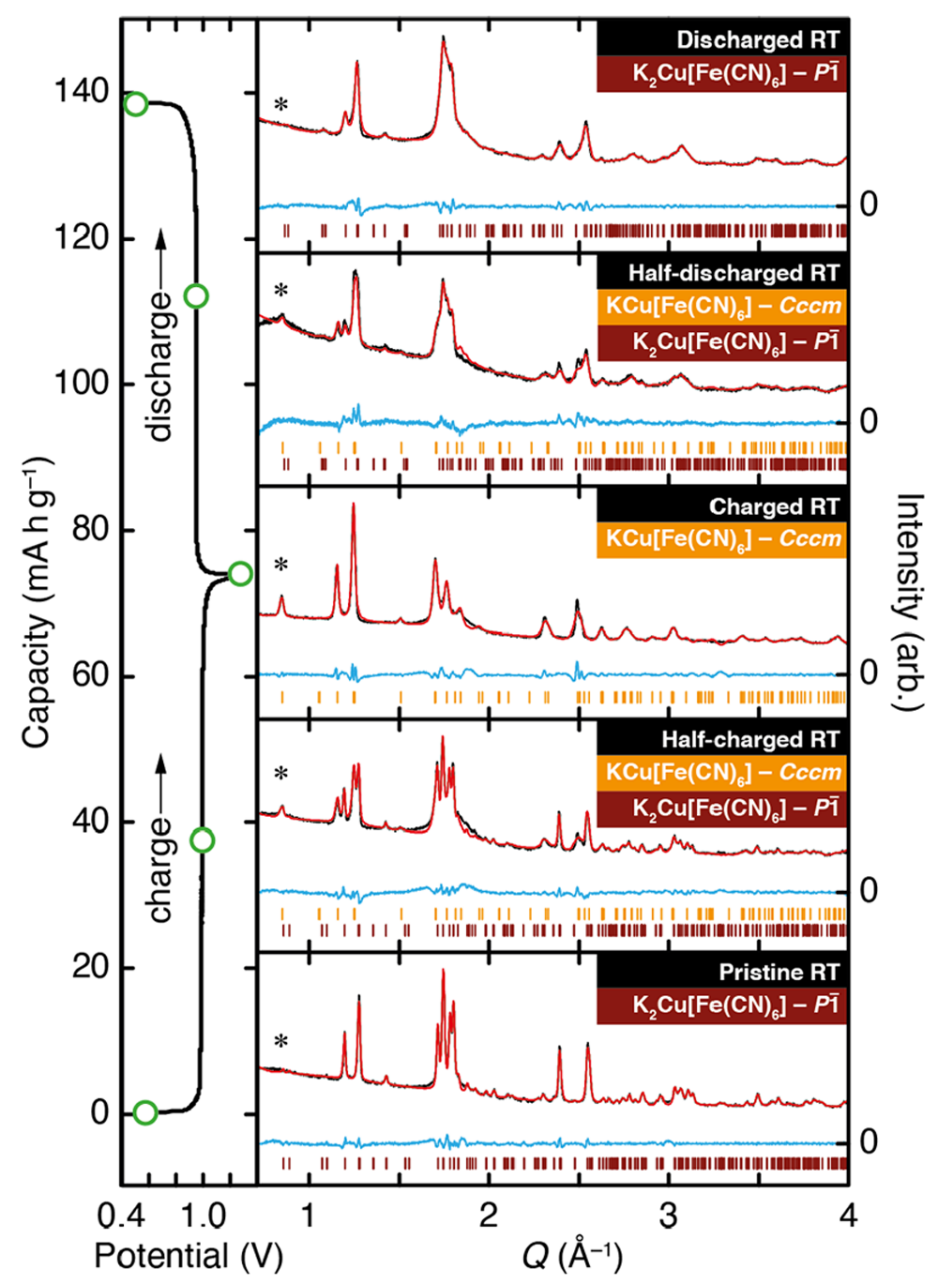
*Ex situ* X-ray powder diffraction measurements for K_2–*x*_Cu[Fe(CN)_6_] samples taken from different key points in the first charge/discharge cycle. Data are shown as black points, fits are shown as red lines, and the difference (data – fit) is shown as blue lines. Tick marks show the allowed reflection positions for both *P*1̅ (dark red) and *Cccm* (gold) phases. The reflections marked with an asterisk are sensitive to the X_5_^+^ cation order.

We tested this hypothesis by carrying out a series of *ex situ* powder X-ray diffraction measurements on K_2_Cu[Fe(CN)_6_] samples taken at five key points in the first charge/discharge cycle. Our results, which we proceed to explain, are shown in Figure 7. The first measurement was taken prior to charging and is entirely consistent with the *P*1̅ structure type determined in our higher-resolution synchrotron X-ray study discussed above. Halfway through the first charge, a particularly complex diffraction pattern is observed that then simplifies considerably at the point of full charge. That third measurement, which on the basis of our electrochemical results corresponds to the approximate composition of KCu[Fe(CN)_6_], can indeed be accounted for by a single phase with *Cccm* symmetry. The complex intermediate diffraction pattern at half-charge can then be fitted using a two-phase *P*1̅ /*Cccm* model, with intensities taken from the pristine and fully charged patterns. Our measurements taken on discharge are similar in their implications. The final diffraction pattern is again characteristic of the K_2_Cu[Fe(CN)_6_] *P*1̅ structure type, albeit with significantly broadened reflections, and the pattern taken at the half-discharge point can again be fitted using a two-phase model. Full details of our fitting procedure and results of the various refinements are given in the SI.

Just as K_2_Cu[Fe(CN)_6_] responds to thermal activation by switching off the K-ion slides and ascending from *P*1̅ to *Cccm* symmetry, so too does electrochemical K-ion extraction have the same effect.

A peculiarity of both *P*1̅ and *Cccm* structures to which we have already alluded is that they contain two crystallographically distinct K-ion sites. This reflects the X_4_^+^ cation order we know to be present. In the case of the *Cccm* KCu[Fe(CN)_6_] (fully charged) phase, there is clear evidence for K^+^/vacancy order: the emergence of diffraction intensity near *Q* = 0.85 Å^–1^ is characteristic. A structural model with equal K-ion occupancies on the two crystallographically distinct sites gives no appreciable intensity at this position; after all, this is why there is no intensity here for the fully potassiated phase. Hence, there is selective extraction of K^+^ ions from just one subset of the K-ion channels in K_2_Cu[Fe(CN)_6_]. Unfortunately, our data are not of sufficiently high quality to allow robust Rietveld refinement of the corresponding occupancies.

DFT calculations also reflect this preference for cooperative K-ion extraction. Starting with the relaxed *P*1̅ structure described above, we removed in turn all possible combinations of two of the four potassium ions in its unit cell and then rerelaxed the corresponding structures. The two configurations with rodlike (X_4_^+^) K-ion/vacancy order relaxed to lower energies (∼6 meV/atom) than other combinations. Interestingly, in the resulting KCu[Fe(CN)_6_] structures there persisted some off-centering of the K^+^ ions, which reduced the symmetry from *Cccm* to *P*1̅. This suggests that the *Cccm* structure type we observe may be unstable with respect to a slide distortion at 0 K; presumably, it is simply that the critical temperature at which the distortion occurs is below room temperature. This is the same instability that we have observed (in reverse) when heating the fully potassiated phase, for which the critical temperature is understandably much higher.

As a final point, we note that the increased peak broadening observed on discharge is probably due to a combination of particle size reduction and also domain formation during symmetry lowering. We comment also that it is not our intention here to investigate fully the cycling capacity of K_2_Cu[Fe(CN)_6_], nor the effect of different electrolytes, nor the potential differences between *in situ* and *ex situ* observations;^50^ we expect to follow up on these aspects in a future study. We note simply that the initial capacity observed (73.8 mA h g^–1^) certainly compares favorably against that of the better known K_0.71_Cu[Fe(CN)_6_]_0.72_ phase (59.1 mA h g^–1^).^18^

## Conclusion

**Figure 8:**
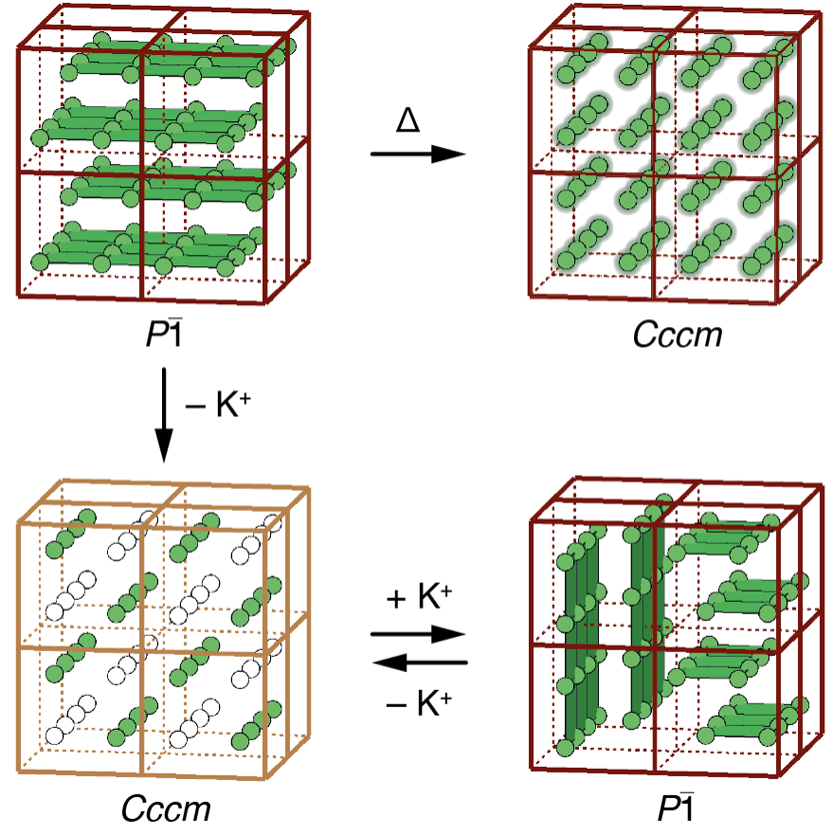
Schematic representation of the structural transformations in K_2_Cu[Fe(CN)_6_] that take place as a function of temperature and electrochemical cycling. On heating, increased K-ion displacements (shown here as blurred green spheres) result in “melting” of the K-ion slide distortion and the ascent in symmetry from *P*1̅ to *Cccm*. Likewise, electrochemical extraction of K^+^ from the ambient *P*1̅ phase gives KCu[Fe(CN)_6_] with rodlike A-site vacancy order that again disrupts the slide distortion. Subsequent reinsertion of K^+^ reactivates the K-ion slides, albeit in domains of smaller coherence length than in the pristine sample. Dark-red-colored and gold-colored frames denote the 2+/3+ charge state of Fe.

In summary, we have prepared and characterized the new PBA material K_2_Cu[Fe(CN)_6_]. Its complex triclinic structure arises from the interplay of K-ion slides, octahedral tilts, cooperative Jahn–Teller order, and rodlike K-ion occupational order. Of these various distortions, the K-ion slides are what dominate the structural response of the material. We see this both in terms of the behavior at high temperatures and the structural changes that take place during electrochemical cycling. As esoteric as the various symmetry considerations associated with combining distortions might seem, one very physical consequence is that K-ion extraction proceeds via a two-phase mechanism to give a charged phase with rodlike K-ion order. This implies a specific migration pathway. A schematic representation of these various transformations is given in Figure 8.

At face value, the structural transformations taking place during K-ion (de)insertion in K_2_Cu[Fe(CN)_6_] perhaps seem very much more complicated than the solid-solution cubic-phase behavior of other PBAs. Of course, it is only because the various distortions are ordered in our new material that we can see what is actually going on. There can be no doubt that disordered phases such as K_0.71_Cu[Fe(CN)_6_]_0.72_ exhibit the very same types of distortions we discuss here—it is simply that these distortions are correlated only over comparatively smaller distances. Nevertheless the symmetry arguments we apply to our ordered phase will still affect the local behavior of these disordered materials. We now know there must be strong coupling between, e.g., local Jahn–Teller order and the orientation of vacant A-site channels in K_0.71_Cu[Fe(CN)_6_]_0.72_, even if there is no obvious signature of this in the average crystal structure. Our identification of the key symmetry-lowering mechanisms at play also simplifies the use of pair distribution function measurements to characterize the local structure and its evolution in these important and useful cathode materials.^51,52^

As a final point, we comment that the A-site slide degree of freedom—revealed here as the key distortion mode in K_2_Cu[Fe(CN)_6_]—may turn out to be an effective ingredient in designing ferroelectric PBAs. In conventional perovskites, antipolar A-site distortions of the same type are induced by the common *Pnma* tilt distortion (e.g., as in SrSnO_3_). It was shown in ref (53) that the right kind of A-site compositional order in any such system would necessarily give rise to a polar phase. The polarization direction and tilt sense are linked by a trilinear coupling term in the free energy expansion, and hence the polarization might be reversed in an applied field by inverting the sense of octahedral rotation. While the K_2_Cu[Fe(CN)_6_] structure type also contains an A-site cation order, the two types of K^+^ ions are evenly distributed within each sliding plane, which is why this structure is not polar. Nevertheless, the large number of different possible tilt systems and other degrees of freedom accessible to PBAs^22,23^ (and hybrid perovskites, more generally^10,13^) might allow the clever design of other systems in which other kinds of A-site cation orders couple with A-site slides to drive a hybrid improper ferroelectric response.^54^ We intend to revisit this point in a future study of slide distortions in PBAs.

## Supporting Information Available

The following files are available free of charge. The Supporting Information is available free of charge at https://pubs.acs.org/doi/10.1021/acs.chemmater.2c00288.

Additional experimental details, materials, methods, and supplementary discussion, including details of sample characterization by ICP-MS, TGA, and SEM; a full description of powder diffraction refinement approaches and their results; further information regarding DFT calculations; and electrochemical characterization (PDF)
Representative TOPAS input files (ZIP)
