# Supplementary material for: Uncovering the Interplay of Competing Distortions in the Prussian Blue Analogue K2Cu[Fe(CN)6]
Source: Chem Mater. 2022 May 24;34(11):5000–8. doi: 10.1021/acs.chemmater.2c00288 (PMC9202302; doi:10.1021/acs.chemmater.2c00288)
Supplement: Supplementary file 1 — cm2c00288_si_001.pdf [file cm2c00288_si_001.pdf]

Uncovering the interplay of competing distortions in the Prussian blue analogue $K_2Cu[Fe(CN)_6]$

SUPPLEMENTARY INFORMATION

John Cattermull,[a,b] Krishnakanth Sada,[b] Kevin Hurlbutt,[b]
Simon J. Cassidy,[a] Mauro Pasta[b*] and and Andrew L. Goodwin[a*]

[a]Department of Chemistry, University of Oxford, Inorganic Chemistry Laboratory,
South Parks Road, Oxford OX1 3QR, U.K.

[b]Department of Materials, University of Oxford, Parks Road, Oxford OX1 3PH, U.K.

*To whom correspondence should be addressed;
E-mail: mauro.pasta@materials.ox.ac.uk, andrew.goodwin@chem.ox.ac.uk

Contents

1 ICP-MS **3**

2 TGA **5**

3 SEM **6**

4 Powder X-ray diffraction **7**

5 DFT **18**

6 Electrochemistry **21**

7 References **23**

1 ICP-MS

Our $K_2Cu[Fe(CN)_6]$ sample was digested in concentrated HCl and then diluted in 2% nitric acid to give a final Cu concentration of approximately 70 parts per billion (ppb). Calibration for Cu, and Fe compositions was achieved through a series of standard nitrate solutions (Certipur, Merck) of known compositions; we used final concentrations of 0, 1, 2, 5, 10, 20, 40, 60, 80, 100 ppb.

For our sample and for each standard solution, the corresponding Cu and Fe compositions were determined using a Shimadzu ICPMS-2030 spectrometer coupled with a mini torch and an AS-10 autosampler. A helium collision cell was used to remove interfering polyatomic species. Measurements were performed in triplicate, with washing runs carried out between successive measurements. The measured ICP-MS intensities I(Fe), I(Cu) for our series of standard solutions are listed in Table S1; those for our $K_2Cu[Fe(CN)_6]$ sample are given in Table S2, together with the calculated Cu and Fe concentrations.

f_{nom}(M)/ppb	I(Fe 56)/cps	I(Cu 65)/cps
0	26(4)	66.0(6)
1	21.4(4)	11(2)
2	22(2)	17.6(8)
5	38(1)	37(2)
10	64(1)	65.9(3)
20	120.7(4)	125.0(3)
40	227(1)	245.0(8)
60	347(2)	353(1)
80	431(1)	464.6(3)
100	526.2(4)	570.2(7)

Table S1: Average ICP-MS data for our standard solutions.

	Fe 56	Cu 65	ratio
I/cps	298.8(3)	376.6(4)	
$\rho/\mu g\,dm^{-3}$	54.2(3)	64.3(4)	
c/mol dm^{-3}	0.968(6)	0.989(6)	0.979(8)

Table S2: Average ICP-MS data for our $K_2Cu[Fe(CN)_6]$ sample.

2 TGA

The TGA experiment accounted for any mass loss associated with the Al_2O_3 crucible and other instrument components by discounting the mass loss from a blank run with the same experimental conditions. Since the experiment was performed under Argon atmosphere it is likely the mode of decomposition and associated temperatures of certain events are not identical to that elucidated from the variable temperature XRD experiment.

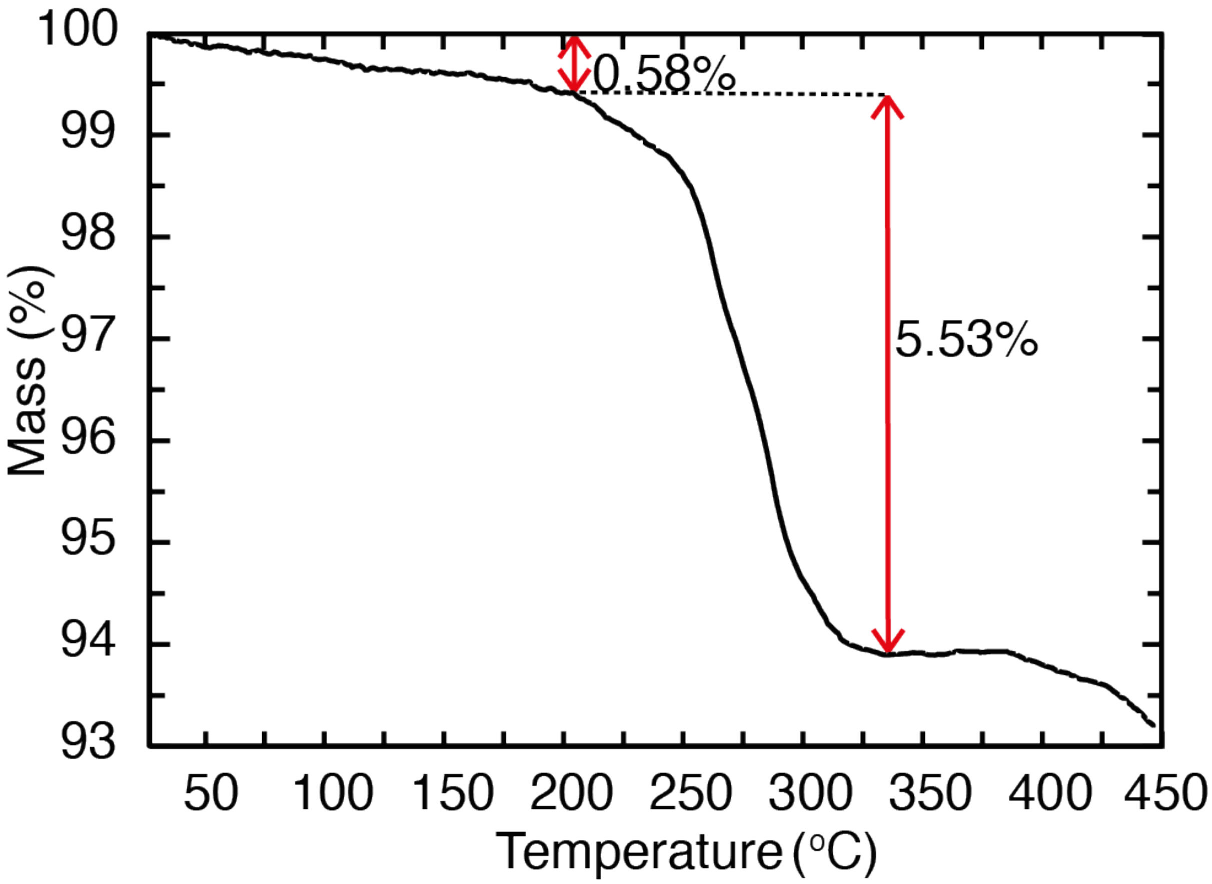

Figure S1: The mass loss of 0.58% up to 203 °C corresponds to 0.11 moles of water per formula unit ($K_{1.96}Cu[Fe(CN)_6]_{0.98} \cdot 0.11H_2O$). The second mass loss event (5.53%) can be attributed to gaseous $(CN)_2$. This mass loss corresponds to 0.37 moles of $(CN)_2$. The theoretical value of 0.5 based on complete reaction of our proposed decomposition pathway would give an expected mass loss of 7.52%.

3 SEM

To probe the morphology of our $K_2Cu[Fe(CN)_6]$ sample, particles were dispersed in ethanol and then mounted on an aluminium stub. These particles were then imaged using a Zeiss Merlin scanning electron microscope (SEM) equipped with a field emission gun, operated at an accelerating voltage of 10 kV and a probe current of 100 pA. A representative image is shown in Fig. S2.

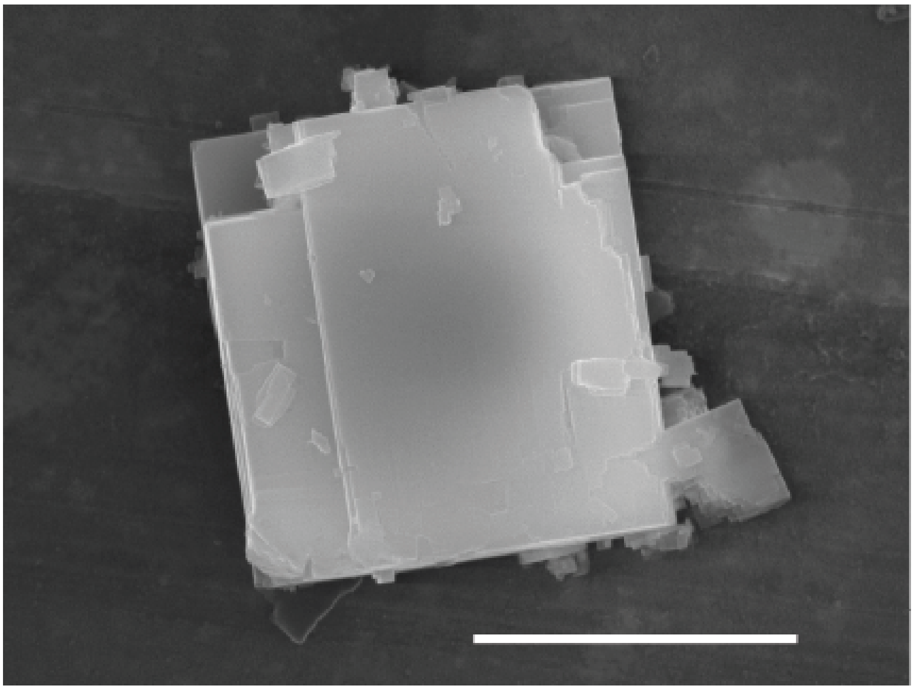

Figure S2: Scanning electron micrograph of a $K_2Cu[Fe(CN)_6]$ particle, scale bar 1 μm.

4 Powder X-ray diffraction

Measurements

Our powder X-ray diffraction data made use of two independent types of measurement: a synchrotron X-ray powder diffraction study at both room and elevated temperatures, and an *ex situ* in-house study of samples extracted during cycling.

The synchrotron powder X-ray diffraction patterns of the sample were collected in capillary transmission geometry using the I11 beamline at Diamond Light Source. A room temperature X-ray diffraction pattern was collected using the Mythen2 Position Sensitive Detector (PSD), two data collections of 5 seconds each were taken at angles 0.25 degrees apart, then summed to account for gaps in the detector coverage.

Measurements between 30 and 450 °C were performed using an FMB Oxford cyberstar hot air blower aimed side-on to the sample at the beam position. Data were collected by continually warming the sample at a rate of 12 $^{\circ}$C min^{-1} while collecting two 4 second scans with the PSD, with 15 second wait times between collections to mitigate beam damage. Due to the side-on nature of the heating it was not feasible to move the capillary position to expose new sample without introducing a temperature hysteresis. No evidence of beam damage was observed in either experimental setup.

Our *ex situ* X-ray powder diffraction measurements of the material were performed in-house on a Rigaku Smartlab diffractometer equipped with a Cu Kα source. The electrode was rinsed with deionised water, and dried in air (70 °C) overnight before mounting the whole electrode onto a sample holder. Each sample was aligned with the source/detector before measurement.

Ambient-temperature data: refinement details

The structural refinement for room temperature $K_2Cu[Fe(CN)_6]$ was performed using a Rietveld refinement in TOPAS software,[S1] in conjunction with distortion modes obtained from ISODISTORT.[S2, S3] The parent cell was a cubic crystal structure of $K_2Cu[Fe(CN)_6]$ in $Fm\bar{3}m$ with cell parameter 10.08 Å, with atomic positions taken from the structural model for copper hexacyanoferrate reported in Ref. S4. The starting values for the strain-mode amplitudes were estimated using a mode decomposition method from a distorted structure found *via* a Pawley refinement of the pattern. K occupancies were allowed to vary as a function of the X_4^+ distortion mode. C–N (1.15 Å), Fe–C (1.9 Å), and Cu–N (2.0 and 2.4 Å) bond distances were restrained, with a relatively high tolerance (0.1 Å) applied. These soft restraints prevented

atoms with a poor scattering contrast from moving to unphysical positions. Note that the Cu Jahn–Teller distortion was robust to removal of the Cu–N bond restraints. Positions of all of the atoms were allowed to refine as a function of the 42 distortion modes generated by a distortion from $Fm\bar{3}m$ to $P\bar{1}$, many of which were minor in contribution, but allowed by symmetry. All Cu, Fe, C, and N atoms were found to have similar thermal displacement parameters when freely refined. So, in order to reduce the number of free parameters, these values were fixed to a single common value in our final refinements. Both K sites were allowed to refine with independent thermal displacement parameters. Finally, an anisotropic peakshape model for a monoclinic unit cell was applied to account for the different strains in different directions that individual crystallites will have, giving a better interpretation of the peak intensities.[S5] Without this peakshape term, the best fit we could obtain gave $R_{wp} = 3.24\%$.

Tables S3 and S4 include selected bond lengths and K-ion environments.

K1 and K2 had the following displacement from the central position (0, 0.5, 0.25) and (0.5, 0, 0.25)

Table S3: Selected bond distances for the room temperature $P\bar{1}$ structure of $K_2Cu[Fe(CN)_6]$ reported in Table 1.

Atoms	Bond distance (Å)
C1–N1	1.17(3)
C2–N2	1.13(3)
C3–N3	1.17(3)
C4–N4	1.14(3)
C5–N5	1.17(3)
C6–N6	1.17(3)
Fe1–C1	1.98(2)
Fe1–C2	1.89(2)
Fe1–C5	1.87(2)
Fe2–C3	1.89(2)
Fe2–C4	1.89(2)
Fe2–C6	1.90(2)
Cu1–N3	2.390(17)
Cu1–N4	2.041(17)
Cu1–N5	2.02(2)
Cu2–N1	2.357(18)
Cu2–N2	2.031(17)
Cu2–N6	2.02(2)

Table S4: Bond lengths for K–CN interactions.

Atoms	d(K1–X) (Å)	d(K2–X) (Å)
K–C1	3.40(2)	3.188(19)
K–C2	3.37(2)	3.73(2)
K–C3	3.20(2)	3.41(2)
K–C4	3.18(2)	3.69(2)
K–C5	3.44(2)	3.07(2)
K–C6	3.39(2)	3.08(2)
K–N1	3.416(16)	3.052(17)
K–N2	3.311(18)	3.72(2)
K–N3	3.146(17)	2.975(16)
K–N4	3.283(18)	3.76(2)
K–N5	3.255(17)	3.308(17)
K–N6	3.54(2)	3.122(19)

respectively:

$$\mathbf{u}_{\mathrm{K1}} = 0.038\mathbf{a} - 0.066\mathbf{b} + 0.000\mathbf{c}, \quad (1)$$

$$\mathbf{u}_{\mathrm{K2}} = -0.008\mathbf{a} + 0.055\mathbf{b} + 0.002\mathbf{c}. \quad (2)$$

These correspond to displacement magnitudes $|\mathbf{u}_{\mathrm{K1}}|$ = 0.534(4) Å, and $|\mathbf{u}_{\mathrm{K2}}|$ = 0.403(4) Å.

Variable-temperature data: refinement details

Our refinement strategy differed for two separate temperature regimes.

The first strategy—used for data collected in the temperature range 30–246 °C—consisted of a sequential Rietveld refinement with the $P\bar{1}$ $K_2Cu[Fe(CN)_6]$ model, using a distortion mode refinement approach. Starting from the room temperature structure as given in the main text, we allowed the lattice parameters, atomic positions and thermal displacement parameters to refine in the same way as described above for the room temperature structure. Site occupancies were fixed and bond distances were restrained to prevent C and N atoms from moving to unphysical positions.

A second strategy was employed for the temperature range 246–450 °C. The refinement now includes standard atomic position-based Rietveld refinements for the $K_2Fe[Fe(CN)_6]$ and $KCu(CN)_2$ phases as well as a distortion-mode Rietveld refinement for $K_2Cu[Fe(CN)_6]$ in $Cccm$. For the two former cases,

Table S5: Crystallographic parameters for the $Cccm$ structure of $K_2Cu[Fe(CN)_6]$ at 246 °C. Refinement constrained B_{eq} for all C and N sites to be identical.

a (Å)	10.63858(10)			
b (Å)	9.90011(7)			
c (Å)	9.92927(5)			
V (Å^3)	1045.782(13)			
Z	4			
Atom	x	y	z	B_{eq} (Å^2)
C1	0.4201(10)	0.1678(11)	0	4.67(13)
C2	0.25	0.25	0.807(2)	4.67
C3	0.278(2)	0.449(2)	0	4.67
Cu	0.25	0.75	0	1.03(8)
Fe	0.25	0.25	0	3.51(13)
K1	0	0.5	0.25	6.4(3)
K2	0	0	0.25	6.0(3)
N1	0.532(2)	0.204(3)	0	4.67
N2	0.25	0.25	0.6974(17)	4.67
N3	0.8100(16)	0.0672(17)	0	4.67

we refined the lattice parameters, atom coordinates (but not those of C and N in our $K_2Fe[Fe(CN)_6]$ model), and thermal displacement parameters (constraining those for C and N to be the same). For $K_2Cu[Fe(CN)_6]$ an orthorhombic anisotropic peak broadening term was included to account for residual strain broadening accompanying the phase transition from $P\bar{1}$ to $Cccm$.

Crystallographic details for various key structural models obtained in our refinements are given in Tables S5–S7. The full temperature dependence of the unit-cell parameters and key distortion mode amplitudes (where relevant) are given in Tables S8–S11.

Table S6: Crystallographic parameters for the $P2_1/c$ structure of $KCu(CN)_2$ at 307 °C. Refinement constrained B_{eq} for all C and N sites and all both K and Cu sites to be identical.

a (Å)	7.61504(12)			
b (Å)	7.91390(12)			
c (Å)	7.61082(17)			
β (°)	100.8305(13)			
V (Å^3)	450.494(14)			
Z	4			
Atom	x	y	z	B_{eq} (Å^2)
C1	0.322(7)	0.184(5)	0.000(4)	5.4(11)
C2	0.067(6)	0.918(10)	0.232(9)	5.4
Cu	0.1324(7)	0.1205(8)	0.1269(7)	6.38(19)
K	0.3038(9)	0.5651(10)	0.1836(11)	6.38
N1	0.462(4)	0.266(3)	0.004(4)	5.4
N2	0.051(4)	0.829(6)	0.337(7)	5.4

Table S7: Crystallographic parameters for the $P2_1/n$ structure of $K_{1.839(5)}Fe[Fe(CN)_6]$ at 420 °C. Refinement constrained B_{eq} for all non-TM sites to be identical.

a (Å)	10.2615(2)			
b (Å)	7.2520(2)			
c (Å)	7.1893(2)			
β (°)	90.009(14)			
V(Å^3)	535.07(3)			
Z	2			
Atom	x	y	z	B_{eq} (Å^2)
C1	0.068	0.822	0.227	5.34(11)
C2	0.014	0.184	0.164	5.34
C3	0.801	−0.05	0.023	5.34
Fe1	0.5	0	0	2.57(5)
Fe2	0	0	0	2.57
K	0.7562(17)	0.4689(8)	0.0342(7)	5.34
N1	−0.014	0.692	0.341	5.34
N2	−0.035	0.304	0.228	5.34
N3	0.698	0.011	−0.014	5.34

Table S8: Temperature dependence of the unit-cell parameters and slide distortion mode for the $P\bar{1}$ structure of $K_2Cu[Fe(CN)_6]$ in the temperature range 30–246 °C from a single phase refinement.

T (°C)	a (Å)	b (Å)	c (Å)	α (°)	β (°)	γ (°)	V (Å^3)	$\Gamma_5^+(a)$ (%)
31.2	7.0609(10)	7.3390(10)	9.8719(11)	89.8928(11)	89.9090(11)	86.148(19)	510.41(17)	2.760(1)
38.4	7.066(3)	7.338(3)	9.874(3)	89.8961(12)	89.9106(12)	86.14(5)	510.8(5)	2.701(1)
46.5	7.072(4)	7.337(5)	9.877(5)	89.9004(13)	89.9111(13)	86.14(9)	511.3(8)	2.631(2)
51.6	7.075(4)	7.336(4)	9.878(5)	89.9015(13)	89.9115(13)	86.13(8)	511.6(7)	2.593(2)
59.5	7.0807(18)	7.3352(19)	9.881(2)	89.9041(14)	89.9129(14)	86.13(4)	512.0(3)	2.525(1)
66.0	7.086(3)	7.334(3)	9.883(3)	89.9082(14)	89.9137(15)	86.12(5)	512.4(4)	2.465(1)
71.1	7.090(8)	7.333(8)	9.885(9)	89.9083(14)	89.9173(15)	86.12(15)	512.7(14)	2.414(3)
78.0	7.095(6)	7.332(6)	9.887(7)	89.9113(15)	89.9184(15)	86.11(11)	513.1(10)	2.346(2)
84.8	7.1006(18)	7.3304(18)	9.889(2)	89.9141(16)	89.9178(16)	86.11(3)	513.5(3)	2.281(1)
92.3	7.107(6)	7.329(6)	9.891(7)	89.9180(17)	89.9169(18)	86.10(11)	514.0(10)	2.206(2)
99.3	7.112(3)	7.327(3)	9.893(4)	89.9197(19)	89.9171(19)	86.10(6)	514.4(6)	2.132(1)
105.4	7.119(5)	7.325(6)	9.896(6)	89.923(2)	89.917(2)	86.09(10)	514.8(9)	2.050(2)
112.2	7.1249(8)	7.3230(8)	9.8979(9)	89.923(2)	89.919(2)	86.084(15)	515.22(14)	1.966(1)
119.1	7.131(6)	7.321(6)	9.900(7)	89.925(2)	89.919(2)	86.08(11)	515.6(10)	1.884(2)
125.6	7.138(3)	7.318(3)	9.902(3)	89.926(3)	89.918(3)	86.07(6)	516.1(5)	1.791(2)
132.5	7.1449(10)	7.3156(10)	9.9045(11)	89.931(3)	89.914(3)	86.065(18)	516.48(17)	1.694(2)
138.1	7.1514(11)	7.3131(11)	9.9066(12)	89.931(3)	89.918(3)	86.06(2)	516.88(19)	1.604(2)
145.2	7.159(4)	7.310(4)	9.909(5)	89.928(3)	89.921(3)	86.05(8)	517.3(7)	1.495(2)
152.2	7.166(3)	7.307(3)	9.911(3)	89.927(2)	89.920(2)	86.04(6)	517.7(5)	1.395(3)
159.7	7.174(6)	7.304(6)	9.913(7)	89.924(2)	89.921(2)	86.04(11)	518.2(10)	1.286(3)
166.3	7.182(9)	7.301(9)	9.915(10)	89.922(2)	89.927(2)	86.03(17)	518.6(15)	1.181(4)
172.5	7.19(2)	7.30(2)	9.92(2)	89.921(2)	89.926(2)	86.0(4)	519(3)	1.084(5)
178.8	7.195(5)	7.296(5)	9.918(5)	89.917(3)	89.934(3)	86.01(9)	519.3(9)	1.002(4)
185.5	7.20(1)	7.293(10)	9.919(11)	89.915(2)	89.938(2)	86.00(19)	519.7(18)	0.911(5)
192.5	7.207(18)	7.291(18)	9.92(2)	89.917(2)	89.936(2)	86.0(3)	520(3)	0.827(5)
199.2	7.214(6)	7.289(6)	9.922(7)	89.916(3)	89.941(3)	85.98(12)	520.4(11)	0.744(5)
206.7	7.2204(19)	7.2866(19)	9.923(2)	89.917(3)	89.940(3)	85.97(4)	520.8(3)	0.658(6)
213.0	7.225(8)	7.285(8)	9.924(9)	89.914(3)	89.942(3)	85.96(16)	521.1(14)	0.592(7)
219.5	7.231(14)	7.283(14)	9.925(15)	89.920(3)	89.942(3)	85.9(3)	521(2)	0.517(7)
226.3	7.239(9)	7.281(9)	9.926(10)	89.922(3)	89.944(3)	85.93(17)	21.8(16)	0.414(8)
232.1	7.246(2)	7.278(2)	9.927(2)	89.928(3)	89.946(3)	85.92(4)	522.2(4)	0.316(10)
239.3	7.255(2)	7.274(2)	9.928(2)	89.938(3)	89.950(3)	85.90(4)	522.6(4)	0.19(12)
246.1	7.263(12)	7.270(12)	9.930(13)	89.946(3)	89.959(3)	85.9(2)	523(2)	0.07(13)

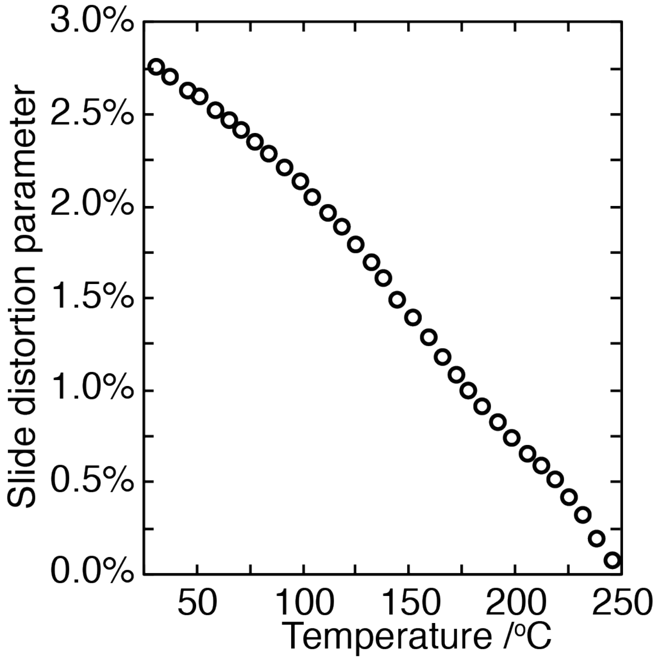

Figure S3: The magnitude of the slide distortion parameter ($\Gamma_5^+(a)$) follows a gradual decline with increasing temperature up to the point at 246 °C where it is zero within error (see Table S8). At this point the pattern is well fit in Ccm, where the slide distortion is forbidden.

Table S9: Temperature dependence of the unit-cell parameters for the $Cccm$ structure of $K_2Cu[Fe(CN)_6]$ in the temperature range 246–347 °C from a mixed phase refinement.

T (°C)	a (Å)	b (Å)	c (Å)	V (Å^3)	Phase fraction (%)
246.1	10.63858(10)	9.90011(7)	9.92927(5)	1045.782(13)	95.8
253.1	10.642(9)	9.902(9)	9.929(10)	1046.3(17)	84.5
259.4	10.643(14)	9.904(14)	9.930(15)	1047(3)	74.4
266.4	10.644(5)	9.905(5)	9.931(5)	1047.0(9)	54.0
273.3	10.644(6)	9.906(6)	9.931(6)	1047.1(11)	42.0
280.0	10.64(3)	9.91(3)	9.93(3)	1047(5)	33.3
287.0	10.644(18)	9.905(18)	9.931(19)	1047(3)	21.0
293.3	10.645(7)	9.904(7)	9.932(8)	1047.1(13)	15.1
300.0	10.646(10)	9.904(10)	9.931(11)	1047.1(19)	13.4
306.7	10.648(8)	9.905(9)	9.932(9)	1047.5(16)	10.4
314.2	10.65(4)	9.91(4)	9.93(4)	1048(7)	8.6
320.5	10.65(2)	9.91(2)	9.93(2)	1048(4)	6.9
326.7	10.65(5)	9.91(5)	9.93(5)	1048(9)	4.7
333.6	10.65(4)	9.91(4)	9.93(4)	1048(7)	3.5
340.2	10.65(4)	9.91(14)	9.93(15)	1050(2)	1.8
347.1	10.6(5)	9.9(5)	9.9(5)	1040(90)	0.4

Table S10: Temperature dependence of the unit-cell parameters for the $P2_1/c$ structure of $KCu(CN)_2$ in the temperature range 253–407 °C

T (°C)	a (Å)	b (Å)	c (Å)	β (°)	Phase fraction (%)
253.1	7.567(12)	7.865(7)	7.544(8)	100.5(10)	1.6
259.4	7.60(3)	7.895(17)	7.59(9)	101.0(10)	0.7
266.4	7.6140(9)	7.9010(11)	7.5905(12)	100.968(11)	5.4
273.3	7.6131(4)	7.9031(5)	7.5931(6)	100.954(6)	9.4
280.0	7.6127(3)	7.9051(3)	7.5967(4)	100.941(4)	12.6
287.0	7.61275(19)	7.9074(2)	7.6001(3)	100.918(2)	17.1
293.3	7.61340(16)	7.90922(18)	7.6041(2)	100.8922(19)	20.2
300.0	7.61437(15)	7.91117(17)	7.6078(2)	100.8609(17)	20.9
306.7	7.61597(16)	7.91322(17)	7.6115(2)	100.8301(17)	21.4
314.2	7.61524(18)	7.9145(2)	7.6147(3)	100.811(2)	19.6
320.5	7.6142(2)	7.9137(3)	7.6156(3)	100.818(3)	15.1
326.7	7.6137(3)	7.9141(3)	7.6170(4)	100.813(3)	13.4
333.6	7.6134(3)	7.9144(4)	7.6179(5)	100.811(4)	11.0
340.2	7.6130(4)	7.9149(5)	7.6189(6)	100.809(5)	8.6
347.1	7.6126(5)	7.9149(6)	7.6188(7)	100.813(6)	7.0
354.1	7.6120(6)	7.9147(7)	7.6187(8)	100.810(7)	5.5
360.9	7.6119(7)	7.9143(8)	7.6191(10)	100.817(8)	4.2
367.0	7.6120(9)	7.9154(9)	7.6203(11)	100.818(10)	3.1
374.1	7.6122(10)	7.9151(11)	7.6210(13)	100.819(12)	2.6
380.2	7.6136(13)	7.9151(13)	7.6223(16)	100.814(15)	1.8
387.3	7.6151(16)	7.9160(16)	7.6224(19)	100.820(19)	1.3
393.2	7.6147(16)	7.915(2)	7.624(2)	100.82(2)	1.0
399.3	7.617(2)	7.918(3)	7.624(3)	100.82(3)	0.6
407.0	7.615(5)	7.895(5)	7.596(6)	100.81(6)	0.8

Table S11: Temperature dependence of the unit-cell parameters for the $P2_1/n$ structure of $K_2Fe[Fe(CN)_6]$ in the temperature range 246–420 °C

T (°C)	a (Å)	b (Å)	c (Å)	β (°)	Phase fraction (%)
246.1	10.095(7)	7.307(3)	7.197(4)	89.69(4)	4.2
253.1	10.095(10)	7.26(2)	7.22(2)	89.9(6)	13.9
259.4	10.105(15)	7.248(9)	7.128(9)	90.2(6)	24.9
266.4	10.090(4)	7.250(3)	7.104(2)	90.1(2)	40.6
273.3	10.090(2)	7.2594(15)	7.0988(13)	90.05(12)	48.6
280.0	10.0929(11)	7.2619(9)	7.0998(8)	90.05(6)	54.1
287.0	10.1006(7)	7.2637(6)	7.1008(5)	90.04(4)	61.9
293.3	10.1100(6)	7.2647(5)	7.1027(4)	90.03(3)	64.7
300.0	10.1181(6)	7.2647(4)	7.1054(4)	90.03(3)	65.7
306.7	10.1267(5)	7.2643(4)	7.1086(3)	90.03(3)	68.2
314.2	10.1365(6)	7.2650(4)	7.1119(3)	90.02(3)	71.8
320.5	10.1486(5)	7.2659(3)	7.1150(3)	90.077(17)	78.0
326.7	10.1597(4)	7.2674(3)	7.1184(3)	90.147(9)	81.9
333.6	10.1702(4)	7.2682(3)	7.1223(3)	90.177(7)	85.4
340.2	10.1796(4)	7.2693(3)	7.1258(2)	90.182(6)	89.6
347.1	10.1885(3)	7.2692(3)	7.1295(2)	90.181(6)	92.6
354.1	10.1966(3)	7.2694(2)	7.1334(2)	90.152(6)	94.5
360.9	10.2041(3)	7.2697(2)	7.1369(2)	90.133(6)	95.8
367.0	10.2115(3)	7.2689(2)	7.14132(19)	90.094(8)	96.9
374.1	10.2185(3)	7.2682(2)	7.14560(19)	90.061(9)	97.4
380.2	10.2251(2)	7.2666(2)	7.15023(18)	90.027(11)	98.2
387.3	10.2320(2)	7.26477(19)	7.15535(17)	90.007(11)	98.7
393.2	10.2382(2)	7.26236(18)	7.16080(17)	89.987(11)	99.0
399.3	10.2439(2)	7.26018(17)	7.16620(17)	89.983(11)	99.4
407.0	10.25091(19)	7.25702(17)	7.17413(17)	89.979(10)	99.2
413.7	10.2566(2)	7.25552(18)	7.18053(18)	89.988(11)	100.0
420.0	10.2615(2)	7.2520(2)	7.1893(2)	90.009(14)	100.0

Ex situ data: refinement details

X-ray powder diffraction patterns from *ex situ* samples were refined using a Pawley refinement approach, since the quality of the diffraction pattern was insufficient for quantitative analysis. We started by fitting the peak intensities for those patterns corresponding to phase-pure samples (*i.e.* full charge or discharge). For the patterns containing two phases, we fixed the reflection intensities to those values determined in that first round of Pawley refinements, but allowed the phase fractions, unit-cell parameters, and peak-shape parameters to refine. The pristine sample was refined using fixed lattice parameters from the RT cif so there are no uncertainties given. No anisotropic peakshape model was used. A summary of the various unit-cell parameters determined in these refinements is given in Table S12.

Table S12: Unit cell parameters for *ex situ* phases

Phase	a (Å)	b (Å)	c (Å)	α (°)	β (°)	γ (°)	V/Z (Å^3)
Pristine $P\bar{1}$	7.056	7.340	9.870	89.8895	89.9024	86.15	255.075
Half charge $P\bar{1}$	7.0716(4)	7.3472(5)	9.8847(6)	89.987(15)	90.002(13)	86.174(5)	256.22(3)
Half charge $Cccm$	10.8616(18)	10.047(2)	10.0715(11)	90	90	90	274.78(8)
Full charge $Cccm$	10.8834(10)	10.039(2)	10.086(2)	90	90	90	275.50(8)
Half discharge $P\bar{1}$	7.1180(9)	7.2968(11)	9.9370(12)	89.604(14)	90.154(13)	86.807(10)	257.65(6)
Half discharge $Cccm$	10.826(3)	10.024(3)	10.061(2)	90	90	90	273.03(13)
Full discharge $P\bar{1}$	7.112(4)	7.306(3)	9.942(8)	89.49(8)	90.09(5)	86.84(5)	257.9(3)

5 DFT

We report in Table S13 the crystallographic details of the DFT-relaxed structure of $K_2Cu[Fe(CN)_6]$ discussed in the main text. While the DFT relaxation process discarded all crystal symmetry, the relaxed structure was found to have $P\bar{1}$ symmetry using the software tool FINDSYM.[S6, S7] The coordinates given have been averaged accordingly. A representation of this relaxed structure is given in Fig. S4 for comparison against the experimentally-determined structure shown in the main text.

As a separate study of the relative energetics of selectively removing one half of the K^+ ions, we calculated the DFT energies for various cells related to that described above for $K_2Cu[Fe(CN)_6]$ but with different combinations of K atoms removed. The corresponding per-atom energies, relative to the lowest-energy arrangement, are given in Table S14. What is clear is that either of the two X-type A-site ordering patterns are energetically preferred.

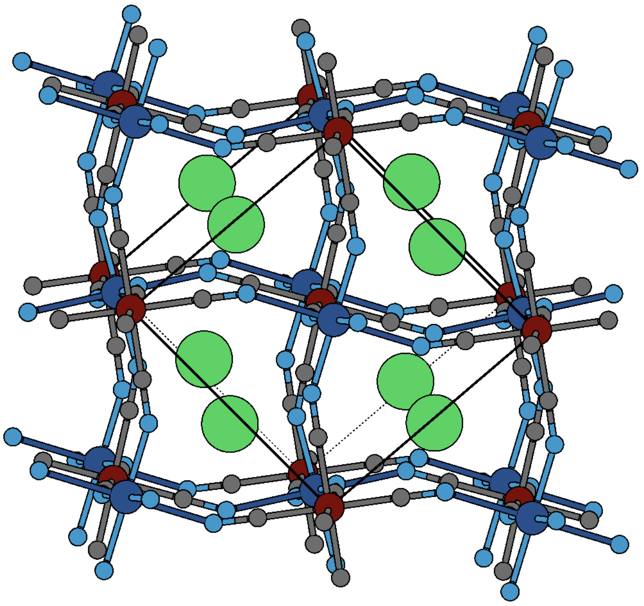

Figure S4: Representation of the 0 K structure of $K_2Co[Fe(CN)_6]$ as determined in our DFT calculations. Atom colours and view direction are as in Fig. 2(b) of the main text.

Table S13: Crystallographic parameters of the DFT structure for $K_2Cu[Fe(CN)_6]$ in $P\bar{1}$

a (Å)	7.09192		
b (Å)	7.33496		
c (Å)	9.84233		
α (°)	89.71256		
β (°)	89.98976		
γ (°)	85.51961		
V (Å^3)	513.54109		
Z	2		
Atom	x	y	z
K1	−0.04568	0.57962	0.24751
K2	0.48863	0.05193	0.24986
Cu1	0	0	0.5
Cu2	0.5	0.5	0
Fe1	0	0	0
Fe2	0.5	0.5	0.5
C1	0.78684	0.86662	0.03239
C2	0.15359	0.77992	−0.01705
C3	0.36641	0.29166	0.53582
C4	0.72322	0.34468	0.48492
C5	0.04750	0.00178	0.18816
C6	0.54119	0.52033	0.68834
N1	0.65694	0.78235	0.05421
N2	0.25116	0.64453	0.97318
N3	0.28374	0.16378	0.55996
N4	0.85856	0.24369	0.47884
N5	0.07259	−0.00022	0.30516
N6	0.55984	0.53296	0.80557

Table S14: Relative DFT energies for different candidate structural models of $KCu[Fe(CN)_6]$. The K-ion occupancies are given relative to the full $P\bar{1}$ structure of $K_2Cu[Fe(CN)_6]$ described above.

K1a	K1b	K2a	K2b	E_{rel} (meV/atom)	A-site order type
1	1	0	0	0.2	rod
1	0	1	0	6.0	layer
1	0	0	1	6.2	dot
0	1	1	0	5.7	dot
0	1	0	1	6.0	layer
0	0	1	1	0.0	rod

6 Electrochemistry

We report the stability window of our electrolyte discussed in the main text, against the dQ/dV plot of the first cycle of $K_2Cu[Fe(CN)_6]$, as evidence for the validity of our electrochemical measurements [Fig. S5].

The potentiostatic intermittent titration technique (PITT) was employed for the second cycle to show that the system remains biphasic after the first cycle. Voltage steps of 2.1 mV were used with a minimum current that corresponded to C/30 and a maximum time limit of 20 mins. The technique works by applying a voltage step, to which the current responds by jumping up. The current them relaxes over time. Once the minimum current is reached another voltage step is applied. If the current does not reach the minimum within 20 mins, a subsequent voltage step is applied.[S8] A delayed response of the current following each voltage step is symptomatic of a biphasic reaction. The result of this is large gaps between the vertical sections of current curve and a bell-shaped I–t curve, which can be seen clearly in Fig. S6.

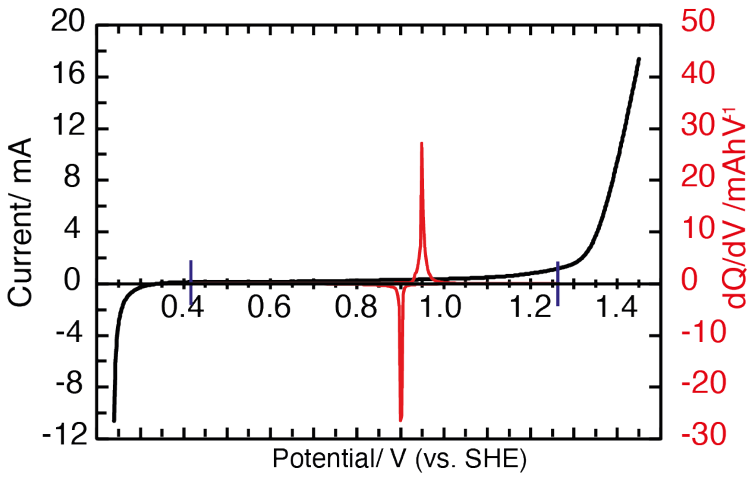

Figure S5: Linear sweep voltammetry (LSV) for the aqueous K_2SO_4 (0.5 M) acidified to pH 1.8 with H_2SO_4. A Hg/Hg_2SO_4 reference in saturated K_2SO_4 and a Pt electrodes were used. The potential limits used for galvanostatic cycling studies are shown in dark blue. A plot of the dQ/dV of the first cycle for $K_2Cu[Fe(CN)_6]$ is overlayed in red.

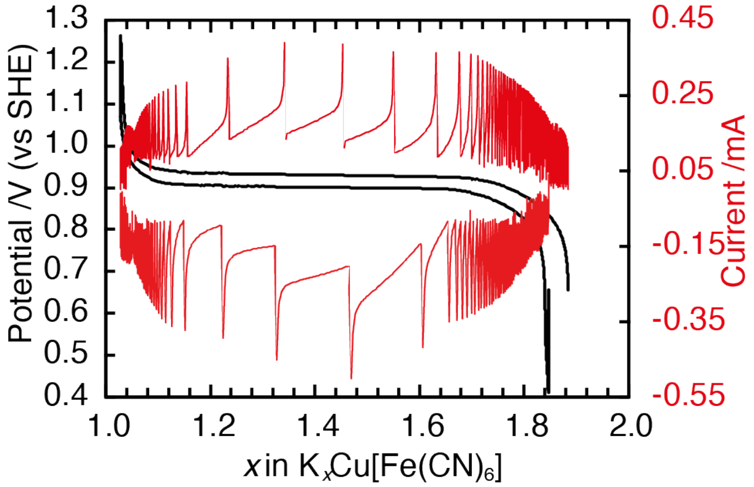

Figure S6: PITT shown in a plot of potential and current against x in $K_2Cu[Fe(CN)_6]$ calculated from the capacity. The cycle starts at x = 1.88

7 References

(S1) A. A. Coelho, *TOPAS-Academic, version 6 (computer software)*, Coelho Software, Brisbane.

(S2) Stokes, H. T.; Hatch, D. M.; Campbell, B. J. *ISODISTORT*, ISOTROPY Software Suite, iso.byu.edu.

(S3) Campbell, B. J.; Stokes, H. T.; Tanner, D. E.; Hatch, D. M. ISODISPLACE: A web-based tool for exploring structural distortions. *J. Appl. Cryst.* **2006**, 39, 607–614.

(S4) Ojwang, D. O.; Grins, J.; Wardecki, D.; Valvo, M.; Renman, V.; Häggström, L.; Ericsson, T.; Gustafsson, T.; Mahmoud, A.; Hermann, R. P.; Svensson, G. Structure Characterization and Properties of K-Containing Copper Hexacyanoferrate. *Inorg. Chem.* **2016**, *55*, 5924-5934.

(S5) Stephens, P.W.; *J. Appl. Cryst.* **1999**, *32*, 281-289.

(S6) Stokes, H. T.; Hatch, D. M.; Campbell, B. J. *FINDSYM*, ISOTROPY Software Suite, iso.byu.edu.

(S7) Stokes, H. T.; Hatch, D. M. Program for Identifying the Space Group Symmetry of a Crystal. *J. Appl. Cryst.* **2005,** *38*, 237-238.

(S8) Zhu, Y.; Gao, T.; Fan, X.; Han, F; Wang, C. Electrochemical Techniques for Intercalation Electrode Materials in Rechargeable Batteries. *Acc. Chem. Res.* **2017**, *50*, 1022-1031.
